# Flipping the switch on the hub cell: Islet desynchronization through cell silencing

**DOI:** 10.1371/journal.pone.0248974

**Published:** 2021-04-08

**Authors:** Janita P. Hogan, Bradford E. Peercy

**Affiliations:** Department of Mathematics & Statistics, University of Maryland Baltimore County, Baltimore, Maryland, United States of America; The University of Michigan, UNITED STATES

## Abstract

Pancreatic *β* cells, responsible for secreting insulin into the bloodstream and maintaining glucose homeostasis, are organized in the islets of Langerhans as clusters of electrically coupled cells. Gap junctions, connecting neighboring cells, coordinate the behavior of the islet, leading to the synchronized oscillations in the intracellular calcium and insulin secretion in healthy islets. Recent experimental work has shown that silencing special hub cells can lead to a disruption in the coordinated behavior, calling into question the democratic paradigm of islet insulin secretion with more or less equal input from each *β* cell. Islets were shown to have scale-free functional connectivity and a hub cell whose silencing would lead to a loss of functional connectivity and activity in the islet. A mechanistic model representing the electrical and calcium dynamics of *β* cells during insulin secretion was applied to a network of cells connected by gap junctions to test the hypothesis of hub cells. Functional connectivity networks were built from the simulated calcium traces, with some networks classified as scale-free, confirming experimental results. Potential hub cells were identified using previously defined centrality measures, but silencing them was unable to desynchronize the islet. Instead, switch cells, which were able to turn off the activity of the islet but were not highly functionally connected, were found via systematically silencing each cell in the network.

## Introduction

Dysregulation of blood glucose can lead to diabetes mellitus and its various complications including cardiovascular disease, chronic kidney disease, and stroke. Consequently, the health of the pancreatic islet, which secretes hormones responsible for maintaining glucose homeostasis, is paramount. Located in the pancreas, the islets of Langerhans primarily consist of *β* cells, representing about 60% of the cells in human islets [1]. These cells secrete insulin into the bloodstream assisting mainly liver, adipose, and skeletal muscle cells with the uptake of glucose from the blood. Since dysfunction in insulin secretion has been linked to type 2 diabetes [2], a further understanding of this phenomena may provide crucial insight into the disease.

Observed in mouse and human islets [3], the electrical activity of *β* cells in an islet, to which insulin secretion is tied [4], undergoes both fast (period of tens of seconds) and slow (period of several minutes) oscillations in response to a glucose stimulus. While *β* cells are heterogeneous in their response, their behavior is coordinated within the islet through gap junctions (Connexin36), which connect the cytosols of neighboring cells allowing ions to diffuse from one cell to another [5–7]. In elevated levels of glucose, this leads to synchronized oscillations, attributed to electrical waves traveling through gap junctions. This is supported by the disappearance of synchronization and electrical waves in gap junction knockout islets [8]. Some sub-regions within the islet were found to be more effective at triggering activity, where the more active cells were co-localized [9]. Subpopulations of *β* cells with differing functions are beginning to be revealed within the islet through studying biomarkers, allowing for further investigation of *β* cell heterogeneity [10]. A loss of coordinated activity has been observed in human islets with type 2 diabetes [11–13], and thus is a focus of much research.

Using optogenetics to activate transfected light sensitive halorhodopsin chloride pumps that hyperpolarize a cell, mouse islets were desynchronized with the silencing of certain *β* cells, called hub cells [14]. To investigate the *β* cell network, functional connectivity graphs, often used in computational neuroscience [15], were created based on calcium trace comparisons. The functional connectivity networks for these islets were scale-free, which were interpreted as having hub-follower scheme in the islet [16]. The hub cells were both (a) highly functionally connected and (b) essential for maintaining oscillations in the islet. They had more hyperpolarized mitochondria, indicative of increased ATP generation, and were more metabolically active due to increased glucokinase protein expression levels [14].

In a computational model to achieve desynchronization with silencing, predetermined “hub” cells required increased glucokinase flux and preferential gap junction coupling while the cellular properties of the nonhub cells were chosen to be below the threshold for activity [17]. However, silencing around 2-6% of the islet (with 10% of cells in the islet as hubs) was necessary to attain the same level of desynchronization as seen experimentally. In addition, the functional connectivity networks were not found to be scale-free. In another study, the presence of hub cells in a computational islet lowered the correlations in cell activity for spiking and fast bursting regimes, while it increased the correlated behavior in slow bursting islets [18]. The difference was explained by the synchronization of the glycolytic oscillator due to metabolic coupling.

Another technique used to study the desynchronization of islets is photoablation, which involves a laser irradiating cells. In zebrafish islets, leader cells, defined as the first to respond to a glucose stimulus, were photoablated, resulting in a notable loss of activity in the islet compared to the minimal differences for the photoablation of follower cells [19]. Granger causality analysis [20] used on the leader cells of mouse islets, which were found to be highly functionally connected, confirmed the predicted causal relationship between the leader and follower cells. A discussion and figure (S10 Fig in S2 Appendix) on the impact on hyperpolarization versus ablation in our simulations is in the S2 Appendix.

The objective of this study is to further investigate the ideas of hub cells, functional connectivity, and desynchronization within the islet through computational methods. A simple model for the individual cells is used to represent the electrical and calcium activity of each *β* cell connected in a hexagon closest packing lattice. Various graph theory measures were applied to the network to study specific characteristics found by Johnston et al. [14]: a scale-free functional connectivity network, a loss of functional connectivity with hub cell silencing, and desynchronization with hub cell silencing. While we were unable to find cells that satisfied all three properties, we did find **switch cells**, whose silencing leads to the loss of activity within the islet, but which have no relation to functional connectivity.

## Methods

In order to computationally investigate hub cells, a mechanistic model for the electrical activity of a single *β* cell during insulin secretion is needed, which is then applied to a network structure. The resulting calcium traces are then transformed into a functional connectivity network, upon which various measures are utilized.

### Model equations, heterogeneity, & islet structure

As pancreatic *β* cells are electrically excitable, their activity can be simulated with models similar to the Hodgkin-Huxley model for neurons. For cell *i* in the *β* cell network, there are four differential equations that describe its electrical and calcium dynamics:

$$\begin{array}{c} -C_{M}\frac{dV_{i}}{dt}=I_{K(ATP)}\left(V_{i}\right)+I_{Ca}\left(V_{i}\right)+I_{K}\left(V_{i},n_{i}\right)+I_{S}\left(V_{i},s_{i}\right)+I_{Coup}\left(V_{i}\right)+I_{Cl}\left(V_{i}\right) \end{array}$$ (1)

$$\begin{array}{c} \frac{dn_{i}}{dt}=\frac{n_{\infty }\left(V_{i}\right)-n_{i}}{\tau_{n}} \end{array}$$ (2)

$$\begin{array}{c} \frac{ds_{i}}{dt}=\frac{s_{\infty }\left(V_{i}\right)-s_{i}}{\tau_{s}} \end{array}$$ (3)

$$\begin{array}{c} \frac{dc_{i}}{dt}=f[-\alpha I_{Ca}\left(V_{i}\right)-k_{Ca}c_{i}]. \end{array}$$ (4)

There are four main ionic currents in the single cell model selected for this study [21]: an ATP/ADP ratio dependent potassium current (*I*_*K*(*ATP*)_) whose closing begins the process of insulin secretion, a voltage-dependent calcium current (*I*_*Ca*_) that leads to the further depolarization of the cell’s membrane, a voltage-gated potassium current (*I*_*K*_) which mediates spike repolarization and a slow inhibitory potassium current (*I*_*S*_) that is responsible for transitions between the active and silent phases of bursting. The full model, with all functional forms, can be seen in the S1 Appendix. For this study, the last two currents were included in the voltage equation shown in Eq (1). The impact of gap junctions was accounted for by the inclusion of a passive coupling current modeled as $I_{Coup}=\sum_{\forall j\in N(i)}g_{c_{ij}}\cdot \left(V_{j}-V_{i}\right)$ for cell *i* where *N*(*i*) represents its nearest neighbors. To simulate optogenetic control over the halorhodopsin chloride pumps in the cell’s membrane, a passive chloride current *I*_*Cl*_ = *g*_*Cl*_ ⋅ *σ* ⋅ (*V*_*i*_ − *E*_*Cl*_) was added where

$$\begin{array}{c} \sigma =\{\begin{array}{c} 1,\text{chloride}\;\text{pumps}\;\text{are}\;\text{activated}\\ 0,\text{otherwise.} \end{array} \end{array}$$

The parameter value for *E*_*Cl*_ was selected such that the passive current would behave in the similar manner as the optogenetically activated pumps. When activated, the cell is sufficiently hyperpolarized and thus its activity is silenced.

Several experimental and computational studies have recently highlighted the importance of *β* cell heterogeneity within an islet [9, 22–24]. Heterogeneity was incorporated into the model by selecting a subset of the cell parameters from truncated (to keep values positive) normal distributions with mean and standard deviation. The distributions for most of the simulations are shown in Table 1; in certain scenarios, a spatial distribution for the parameters was selected, which is described below. The maximal conductance densities were varied as they are related to the number of channels per cell. While individual channel characteristics may also have slight variations, it was assumed that the number of channels was the predominant heterogeneity. The calcium pump rate was drawn from a normal distribution for a similar reason. Most of the parameter values were chosen in accordance to previously published values [25, 26]. The means of the cell parameters were shifted such that a significant number of cells were active when isolated, while the remaining cells were slightly below the threshold for activity. This corresponds to *β* cells in levels of glucose between baseline and elevated (e.g. between about 4mM and 8mM), where it has been shown experimentally that only a fraction of cells will oscillate without coupling [27].

**Table 1:** Cell heterogeneity.

Parameter	Symbol	Mean	SD
Voltage-gated potassium channel conductance	*g*_*K*_	2700 pS	5%
Voltage-gated calcium channel conductance	*g*_*Ca*_	1000 pS	5%
ATP/ADP dependent potassium channel conductance	*g*_*K*(*ATP*)_	100-145 pS	5-10%
Slow inhibitory potassium channel conductance	*g*_*S*_	200 pS	5%
Gap junction conductance	*g*_*c*_	4-200 pS	50%
Calcium pump rate	*k*_*Ca*_	0.2 s^−1^	5%

Heterogeneous parameter values for most islets were selected from Gaussian distributions with the given means and standard deviations.

The gap junctional coupling was made heterogeneous in two ways: coupling strength and number of connections. The gap junctional conductance between a nearest neighbor pair was selected from a normal distribution with a set mean and standard deviation for the islet. Then a binomial distribution would determine if the connection was killed with a probability of 33% to include the possibility of missing gap junction connections physiologically, as had been done in [25]. Initially due to the smaller islet size, the gap junctional conductances were chosen to be low since connectivity across the islet scales with the number of nodes in the network. The same tests were then repeated for islets with stronger coupling. A table of all parameter values can be found in the S1 Appendix.

**Fig 1:**
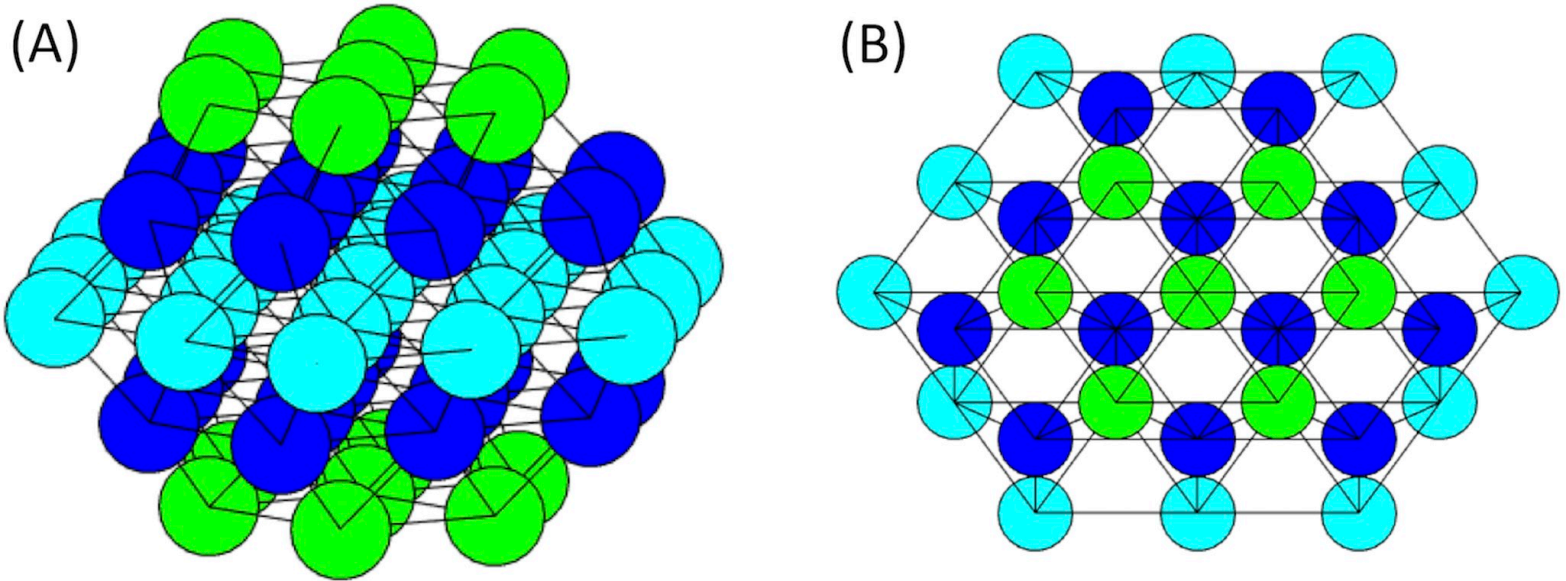
Hexagonal closest packing lattice for islet. The schematic of a hexagonal-closest-packed lattice with *m* = 3 (57 cells). (A) A side view of the islet. (B) The view from above the islet. The colors of the cells are to assist in the differentiation of the layers.

The islet network was simulated as a hexagonal-closest-packing lattice, which allows for more nearest neighbor coupling than the more often used cubic lattice [26]. The main layer of the lattice is a regular hexagon with *m* cells along the edge. There are *m* − 1 alternating layers of irregular and regular hexagons above and below the main layer. An example of this structure can be seen in Fig 1 with *m* = 3, resulting in an islet of 57 cells.

In some cases, the activation cell parameters (*g*_*Ca*_ and *g*_*K*(*ATP*)_) were spatially distributed, motivated by the notion of spatially clustered subpopulations within the islet [9]. A cell in the hexagonal lattice was randomly selected as the orienting cell for the distribution, which had cell parameters following *g*_*K*(*ATP*)_ ∼ *N*(120, 6) and *g*_*Ca*_ ∼ *N*(1100, 55), most likely making it an intrinsically active cell that bursts when uncoupled. For the remaining cells in the islet, the mean value for *g*_*Ca*_ and *g*_*K*(*ATP*)_ depended on their distances from the orienting cell. Cells further from the orienting cell were less likely to be active as the mean values for *g*_*K*(*ATP*)_ increased and *g*_*Ca*_ decreased. The mean values for cell *i* with orienting cell *j* were defined as *g*_*K*(*ATP*)_ = 120 + 10 ⋅ d(*i*, *j*) and *g*_*Ca*_ = 1100 − 50 ⋅ d(*i*, *j*), where d(*i*, *j*) represents the lattice spacing between cells *i* and *j*. Neighboring cells were defined in the lattice as having distance 1. The mean distance between a pair of cells in an *m* = 3 islet was 2.13. The standard deviations for the distributions were 5% of the mean value.

**Fig 2:**
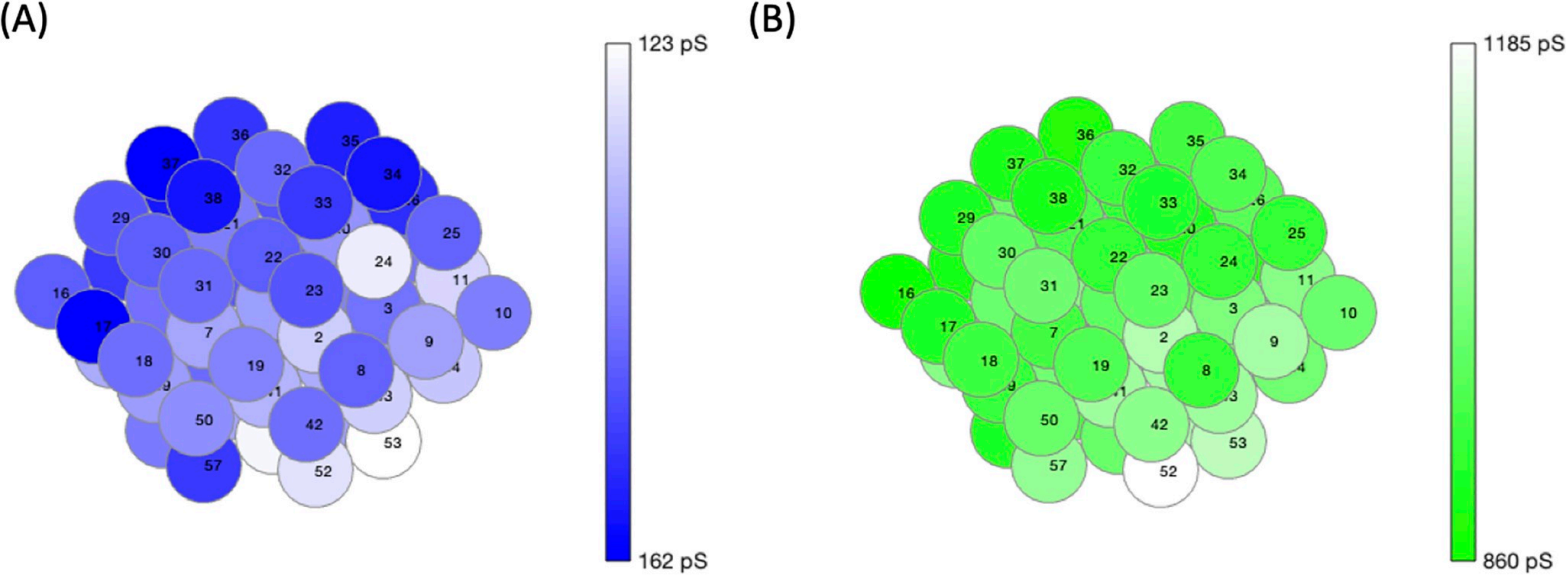
Spatial distributions for *g*_*K*(*ATP*)_ and *g*_*Ca*_. An example of the spatially distributed cell parameters with the orienting cell 53. The darker blue for (A) *g*_*K*(*ATP*)_ and darker green for (B) *g*_*Ca*_ denote the higher conductance values. Cell numbers are for indexing.

An example of the spatially distributed parameters is illustrated in Fig 2 where (A) portrays the *g*_*K*(*ATP*)_ values in the islet and (B) the *g*_*Ca*_ values with the orienting cell chosen as cell 53. The cells further from cell 53 have a darker blue in Fig 2(A), denoting a higher *g*_*K*(*ATP*)_, and a lighter green in Fig 2(B), showing a lower *g*_*Ca*_. While the trend is for the cells to become progressively darker with distance in Fig 2(A) and progressively lighter in Fig 2, these parameter values were drawn from normal distributions centered around cell 53. The slight element of randomness led to some cells such as cell 24 in Fig 2(A) with a lower *g*_*K*(*ATP*)_ value than its neighboring cells.

### Methods of analysis

The solutions to the 57 cell (*m* = 3) system, consisting of 228 coupled differential equations, were numerically computed using a built-in MATLAB differential equation solver (ode45), which implements a variable step-size, variable order Runge-Kutta method. The default settings were used, except for a convergence test. It was run on both a personal MacBook laptop and the high performance computing cluster at UMBC. Various techniques then were used to analyze the network.

#### Functional connectivity

Functional connectivity was first introduced as a method in computational neuroscience for determining regions of the brain with similar electrical behavior, which could imply similar functionality [15]. While various methods of calculating the functional connectivity exist, most focus on correlations of activity between cells in the network. In this study, functional connectivity was used to determine the level of impact cells were having on the islet, which assisted in locating the hub cell. The often used Pearson correlation method [28] assigned functional connections between completely silent cells, which would suggest they were impacting each other’s behavior. However, in the case of two silent cells on opposing ends of the islet, this implication would not be correct. As such, a correlation method with an emphasis on the active phases of cells was selected for this study [29], which was also used by [14].

First, the cells were binarized as active or silent using a calcium threshold of 0.15 *μ*M. The similarity coefficient *C*_*i*,*j*_ for each pair of cells *i* and *j* was calculated using the duration of time cells *i* and *j* were coactive ($T_{i,j}^{*}$) and the active duration for each individual cell (*T*_*i*_ and *T*_*j*_): $C_{i,j}=\frac{T_{i,j}^{*}}{\sqrt{T_{i}T_{j}}}$. The similarity coefficient ranges from 0, when the two cells were not active at the same time, to 1, when the cells were only active together. To ascertain the significance of each connection (i.e. if the connection occurred due to chance), each of the two binarized traces was permuted 10,000 times and the time coactive $T_{i,j}^{\left(k\right)}$ was recalculated each time *k*. A *p*-value was calculated as the probability the permuted coactive time $T_{i,j}^{\left(k\right)}$ is greater than the simulated coactive time $T_{i,j}^{*}$: $p=\frac{\text{card}(\left\{T_{i,j}^{\left(k\right)}:\;\;T_{i,j}^{\left(k\right)}\geq T_{i,j}^{*}\;\;\forall k\right\})}{10,000}$ where **card** is the set cardinality. If *C*_*i*,*j*_ > 0.85 and *p* < 0.01, cells *i* and *j* were defined to be functionally connected. Variation of the threshold for *C*_*i*,*j*_ between 0.8 and 0.9 did not significantly alter results. However a threshold below or above that range led to a much higher or lower, respectively, level of functional connectivity, which did not accurately describe the islet behavior. A new network, separate from that considering only gap junctional weights, arises from the functional connectivity upon which different network measures were calculated.

#### Centralities and hub cells

Centralities are measures of importance in the network that rank the nodes of a graph in order of influence on the network. These measures are often used on functional connectivity, rather than the structural connectivity based on gap junctions and, in neuroscience, synapses [15]. There are various types of centralities such as degree centrality, eigencentrality, betweeness centrality, and closeness centrality that differ in their definition of influence on a network. Johnston et al. [14] used the degree centrality that considers the number of links per node, called the degree of the node, to determine the hub cells they silenced. In *β* cell functional connectivity networks, it can be interpreted as ordering cells by their level of correlated behavior with the other cells in the islet. The cell with the highest degree shares behavior with the greatest number of cells in the islet. We designated this cell as the functional hub cell in the network. A second notion of hubness is related to the desynchronization of the islet. The synchronization hub acts as the organizing center of the islet, synchronizing the activity of the cells in the network. Without the synchronization hub, the islet’s activity is either uncoordinated or lost completely. Our goal was to find a single cell that satisfies both definitions of functional and synchronization hubs, as experimentally reported [14].

#### Scale-free measure

Johnston et al. [14] found their islets containing hub cells had a scale-free functional connectivity network, which has a hub-follower structure [14]. It is defined as a network whose degree distribution asymptotically follows a power law distribution, *P*(*x*) ∼ *x*^−*k*^ with *x*, *k* > 0 where *x* represents the degree of a node [30]. The magnitude of *k* gives an indication of the level of influence of the hub cells in the network, with *k* < 3 denoting a stronger hub-follower structure [31]. This type of network, which has been observed experimentally in islets [14, 16, 19], has very few high degree nodes, many low degree nodes, and is known for its scale invariance. This condition was checked by log transforming the distribution of functional connections and performing a linear regression. A goodness of fit threshold of *R*^2^ > 0.7 was chosen to classify a network as scale-free.

#### Synchronization index

Since a focus of this study was the desynchronization of the islet, a measure was needed to quantify the synchrony of a network. The Fast Fourier Transform was used to determine the most prominent period for each cell trace through the frequency domain representation. The cells of a well synchronized islet should have similar periods. Therefore the mode of cell periods was found for the islet and the synchronization index was the percentage of cells with a period within a few seconds of the mode. Inactive cells, which would have an infinite period, were not considered in the modal calculations, but were included in the total count of cells in the network. Thus, a completely silent islet resulted in a zero synchronization index while a desynchronized islet, which is still active but not synchronized, had a nonzero result. This method was preferred as it did not consider a silent islet to be synchronized, unlike other synchronization measures [25, 26].

**Fig 3:**
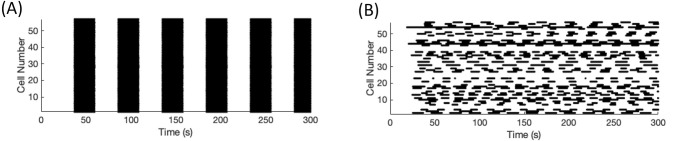
Synchronization of a model islet. Raster plots of cell number versus time in seconds for (A) a completely synchronized and (B) a desynchronized islet. A point is plotted whenever a cell was considered active.

To visually capture the desynchronization, raster plots were used. The calcium traces for all the cells were binarized as active or silent, using the same threshold as for functional connectivity, 0.15 *μ*M. The raster plots capture when each cell is considered active by plotting a point for each time a cell is active, with the cell number along the vertical axis. Fig 3 shows an example of a highly synchronous islet (SI = 100) and a desynchronized islet (SI = 8.77). A significant drop in synchronization, as desired of these networks with hub cell silencing, was considered to be a difference of 40 or greater. Code to generate selected figures may be downloaded from GitHub (https://github.com/bpeercy/SwitchCell).

## Results

With a computational model applied to a network and various network measures, the goal was to satisfy the three criteria laid out in [14]: scale-free functional connectivity, a loss of functional connectivity with hub cell silencing, and a substantial decrease in activity of the islet with hub cell silencing. However, we were unable to achieve all three at one time as scale-free functional connectivity and desynchronization tend to be at odds.

### Scale-free networks do not imply desynchronization with silencing

We first sampled parameter space to find networks with scale-free functional connectivity. Each parameter set was drawn from the normal distributions given in Table 1 with *g*_*K*(*ATP*)_ ∼ *N*(143, 14.3). After an initial simulation was run, the functional connectivity of the network was calculated. If the distribution of functional connectivity was determined to be a power law distribution, the functional hub cells were identified using the degree centrality. Each simulation was then rerun with the silencing of a single functional hub cell and the functional connectivity was recalculated. If the islet was found to have more than one functional hub cell (i.e. multiple cells with the same degree were found to have the highest number of functional connections), each functional hub cell was tested individually. Since another focus of the hypothesis was a loss in functional connectivity with hub cell silencing, parameter sets with a 50% decrease in the number of functional connections were saved to check for desynchronization.

**Fig 4:**
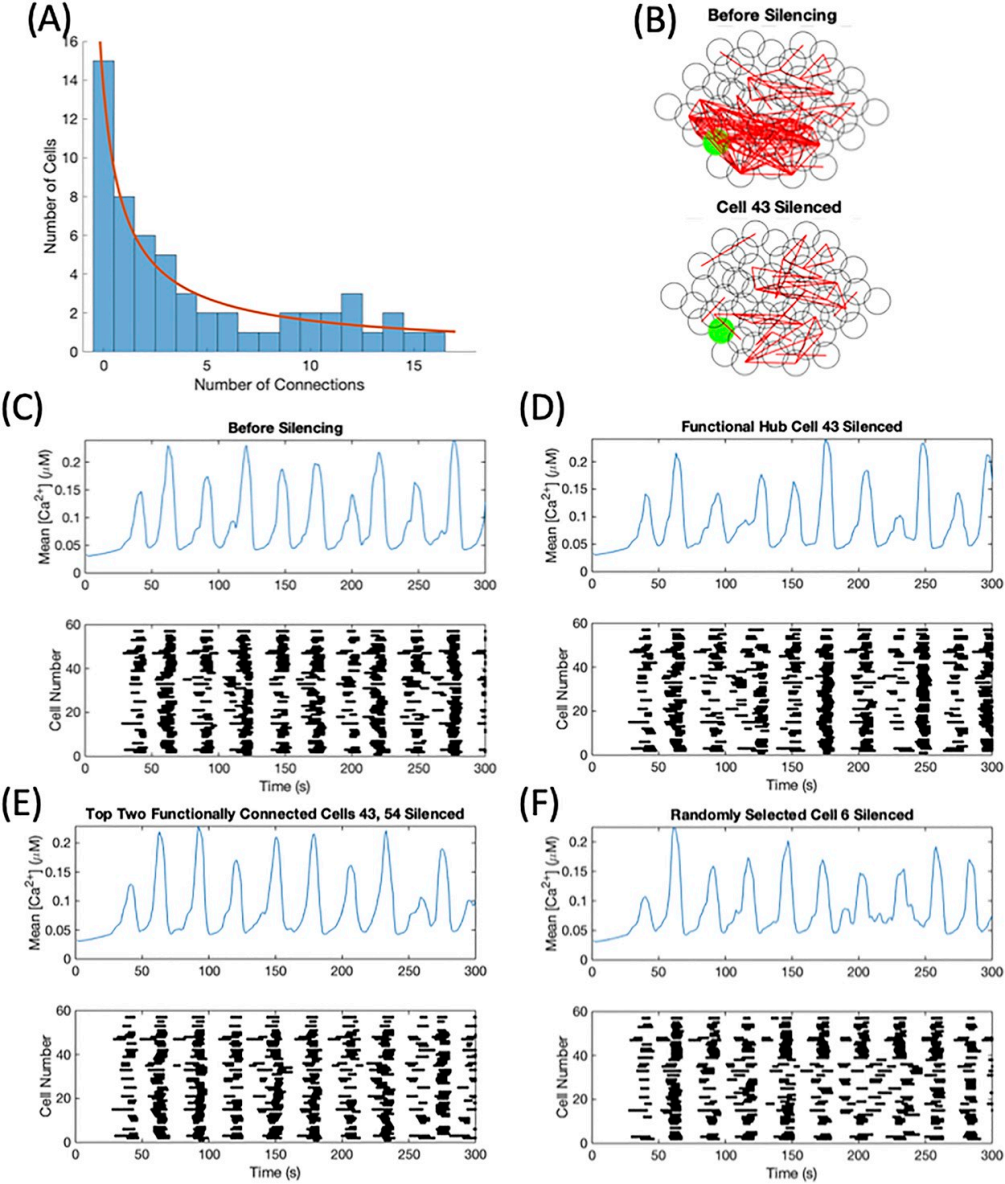
Scale-free connectivity and a loss of functional connectivity with hub cell silencing. This parameter set had *g*_*K*(*ATP*)_ ∼ *N*(143, 14.3) pS and *g*_*c*_ ∼ *N*(4, 2) pS with 33% of connections killed. (A) The distribution of functional connections fitted to a power law with *R*^2^ = 0.77. (B) The functional connectivity graphs before (top panel) and after (bottom panel) the silencing of cell 43, the hub cell. Average calcium time course (top panels) and raster plot (bottom panels) for the islet (C) prior to any silencing, (D) while silencing the functional hub cell, (E) while silencing the top two most functionally connected cells, and (F) while silencing a randomly selected cell in the islet.

For the gap junctional coupling mean set to 4 pS, around 5% of parameter sets (49 parameter sets out of 1000 tested) fit the two criteria of scale-free functional connectivity and a loss of functional connectivity with functional hub cell silencing. An example is shown in Fig 4. The functional connectivity of this parameter set had a distribution fitting a power law with *R*^2^ = 0.77, establishing the network as scale-free by our definition, as shown in Fig 4(A). A plurality of cells had no functional connections while very few had a high number of functional connections. Fig 4(B) illustrates the loss of functional connectivity through silencing. A higher level of connectivity was observed in the initial simulation seen in the top panel, which decreased over 65% once the functional hub cell was silenced depicted in the bottom panel. However, the last objective was not met, displayed in Fig 4(D). The silencing of the functional hub was unable to desynchronize the islet and could not be classified as a synchronization hub. The initial synchrony was low (SI = 49.12) as seen in Fig 4(C), which only decreased to SI = 31.58 when the hub cell 43 was silenced in Fig 4(D). As there was not a substantial desynchronization, the next most functionally connected cell 54 was silenced in addition to functional hub cell 43 shown in Fig 4(E), which was also unable to desynchronize the islet (SI = 29.82). The calcium traces corresponding to Fig 4(C) and 4(D) can be found in S1 Fig in S2 Appendix. As a control, a cell was randomly selected and silenced. As seen in Fig 4(F), the silencing of cell 6 had a similar impact to the silencing of the functional hub cell (SI = 32.58). This lack of drastic desynchronization was common to all the parameter sets fitting the two criterion in this search.

**Fig 5:**
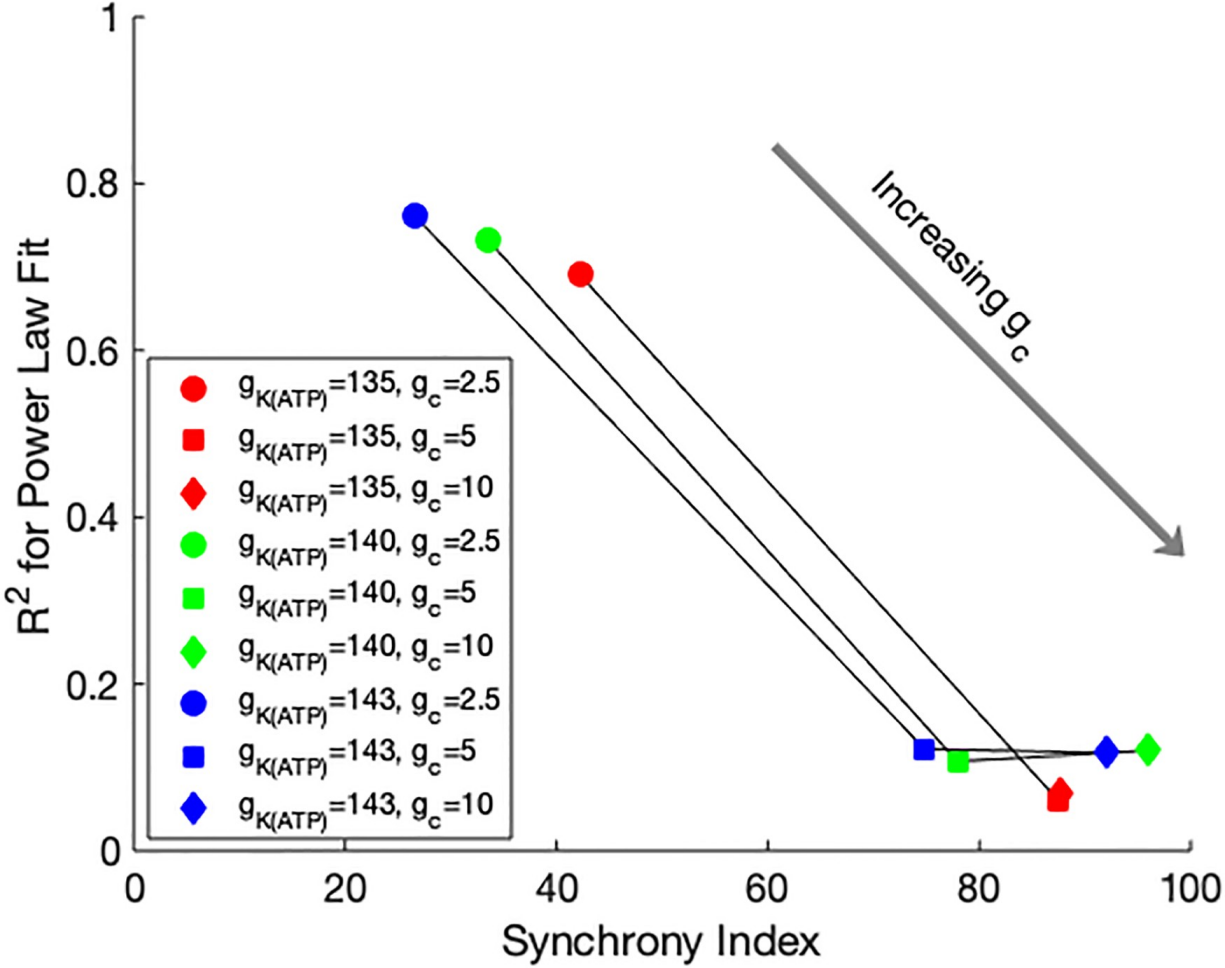
Trade off between scale-free and synchrony. Each data point is the average synchrony index of the islet and average *R*^2^ for the power law fit of the functional connectivity over 100 parameter sets. The means of the parameter distributions are given in the legend. Grey arrow shows the trend of the loss of the scale-free measure and increase in synchrony as *g*_*c*_ increases.

A trade off between scale-free connectivity and islet desynchronization was observed when the above procedure was repeated for a range of *g*_*c*_ values. As shown in Fig 5, when the gap junctional coupling was strengthened, the average scale-free functional connectivity condition drops to inconsequential levels, and cell activity synchronized more strongly, resulting in higher correlations between cells. Each cell had more functional connections and the distributions were less likely to satisfy power law distributions. For smaller gap junctional coupling, fewer cells had correlated activity, which resulted in more parameter sets with scale-free functional connectivity. However, the functional hub cells had less impact on their neighboring cells due to the weaker coupling currents, and minimal changes were observed in the synchronization and functional connectivity when the functional hub cell was silenced.

### Predetermined “hub” cells are not functional hub cells

As the approach of unbiased distribution of cellular and coupling properties in the previous section was unsuccessful in establishing a network with the desired experimental properties including scale-free and desynchronization with hub cell silencing, a different structure for the islet was investigated. A predetermined “hub” cell was placed randomly in the islet with certain parameter values, making it more excitable and more strongly connected based on experimental results [14]. This set up is similar to previous work [17] but with a single predetermined “hub” cell in the islet and a simpler model. The predetermined “hub” cell with *g*_*K*(*ATP*)_ = 100 pS was randomly placed in the islet while the remaining cells had *g*_*K*(*ATP*)_ ∼ *N*(145, 7.25) pS. The *g*_*Ca*_ was still drawn from the distribution given in Table 1. For the given mean values, a Hopf bifurcation, which triggers the oscillatory activity, occurs at *g*_*K*(*ATP*)_ = 133.31 pS, denoting a threshold value for the uncoupled electrical activity. The bifurcation diagram of the single cell can be found in the (S3 Fig in S2 Appendix). The structural connectivity, described by the gap junctional connections, between the predetermined “hub” cell and its nearest neighbors was also set to be twice the strength of the other cells in the islet. The aim of this scheme was to predetermine a cell that could satisfy the definitions of both a functional and synchronization hub.

**Fig 6:**
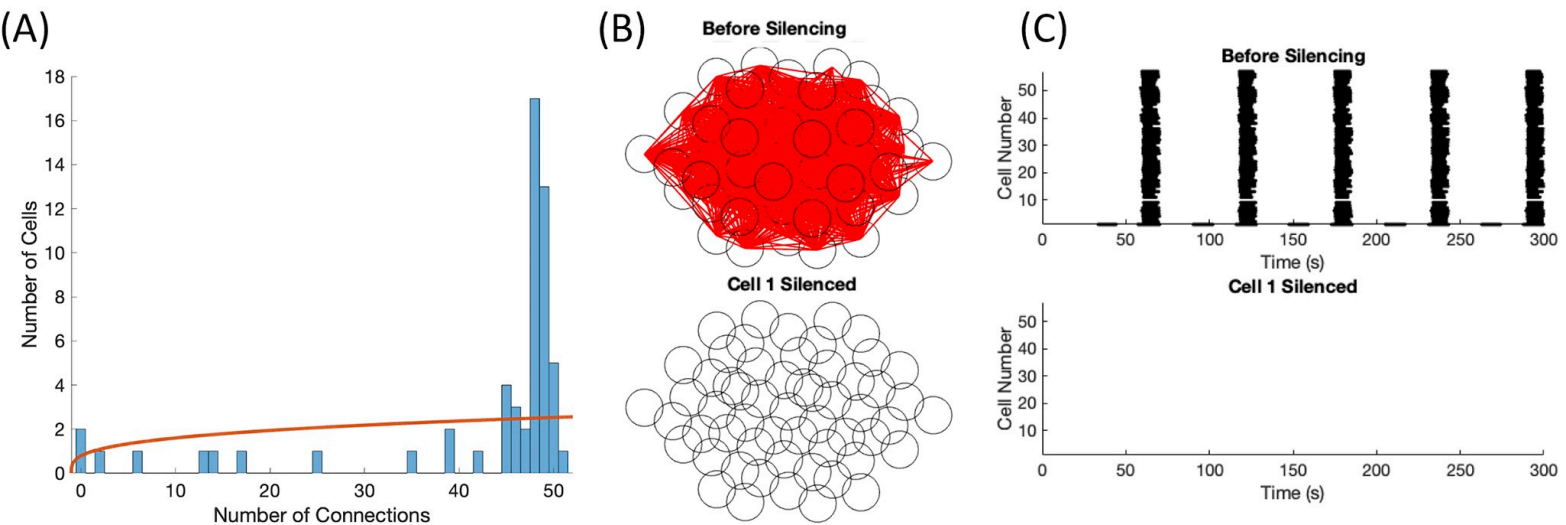
Predetermined “hub” cell. This parameter set had *g*_*K*(*ATP*)_(nonhubs)∼*N*(145, 7.25) pS, *g*_*K*(*ATP*)_(hub) = 100 pS, and *g*_*c*_ ∼ *N*(4, 2) pS. (A) The distribution of functional connections fitted to a power law with *R*^2^ = 0.13. (B) The functional connectivity graphs before (top panel) and after (bottom panel) the silencing of hub cell 1. (C) The raster plots before (top panel) and after (bottom panel) hub cell 1 was silenced.

As expected, the initial synchrony was high (SI = 96.5) for the example shown in Fig 6(C) top panel, since the center cell was able to provide enough stimulus to neighboring cells to activate the rest of the islet. Once the predetermined “hub” cell was silenced, the remaining cells in the islet were no longer recruited into activity and remained silent as well, depicted in Fig 6(C) bottom panel. The calcium traces corresponding to Fig 6(C) can be found in S2 Fig in S1 Appendix. In the initial simulation, almost all of the cells were behaving similarly and thus the majority of the islet was tightly functionally connected as shown in Fig 6(B) top panel. With no activity in the islet post-silencing, the functional connectivity disappeared as it depends on correlated activity in the islet, illustrated in Fig 6(B) bottom panel. As seen in Fig 6(A), the distribution of functional connections did not follow a power law distribution as the fit had *R*^2^ = 0.13.

It is important to note that the predetermined “hub” cell had no functional connections and so it cannot be considered the functional hub cell, leading to a contradiction in the properties described for the hub cell [14]. This has been emphasized by the use of the quotation marks around “hub”. The predetermined “hub” cell was set to be highly active, which led to a difference between the periods of the predetermined “hub” cell and the remaining cells in the islet. As the similarity coefficient, which determined the existence of a functional connection, considered the difference between the duration of each cell’s burst and the duration the two cells are coactive, the difference in periods greatly affected the coefficient. In the example in Fig 6, the predetermined “hub” cell (cell 1) bursts twice for every time the other cells in the islet burst, resulting in the duration coactive between the predetermined “hub” cell and a nonhub cell to be equivalent to the duration of the nonhub cell’s burst and a similarity coefficient $C_{i,j}\leq \frac{1}{\sqrt{2}}$.

Multiple variants of this case were tested in an attempt to get a network with a single functional and synchronization hub, the results of which are summarized in S4 Fig in S2 Appendix. Varying the coupling strength pushed the islet out of the loss of activity regime. As the conductance was increased, the activity of the predetermined “hub” cell was silenced by its neighboring cells, resulting in a silent islet as shown by the calcium time courses in S4(B) Fig in S2 Appendix. Lower conductance values decreased the stimulation from neighboring cells, leading to three possibilities: a completely silent islet, activity only from the predetermined “hub” cell, or a single initial burst among many of the cells in the islet as demonstrated by the raster plots in S4(C) Fig in S2 Appendix. We were unable to find a parameter set where the predetermined “hub” cell had the same bursting rhythm as the other cells under these conditions.

To test the effect of small perturbations, Gaussian noise was added to the voltage dynamics of each cell. There was a threshold for the variation of noise, below which no impact was observed in the raster plots and above which a nonhub cell would become active resulting in an active islet regardless of the activity of the predetermined “hub” cell. The predetermined “hub” cell was randomly placed throughout the islet, instead of only in the center, with minimal difference on the overall islet behavior, pre- and post-silencing. In order to see a little activity in the islet while one predetermined “hub” cell was silenced, a second predetermined “hub” cell was randomly placed in the network. However, instead of the intended effect, the islet remained fully synchronized post-silencing. An example of the two predetermined “hub” cell islet can be seen in the (S5 Fig in S2 Appendix). Thus, this systematic search did not result in any cases satisfying our three constraints.

### Switch cells desynchronize the islet

As using functional connectivity to determine the hub cell did not result in desynchronization with silencing, our focus shifted towards finding a synchronization hub, which desynchronized the islet when silenced. A heterogeneous parameter set was drawn from the distributions in Table 1 such that a fraction of cells were just below the threshold and required only a small stimulus for activity. If the synchrony of the initial simulation was above SI = 60, then each cell in the islet was systematically silenced and the synchrony was measured for the new simulation.

**Fig 7:**
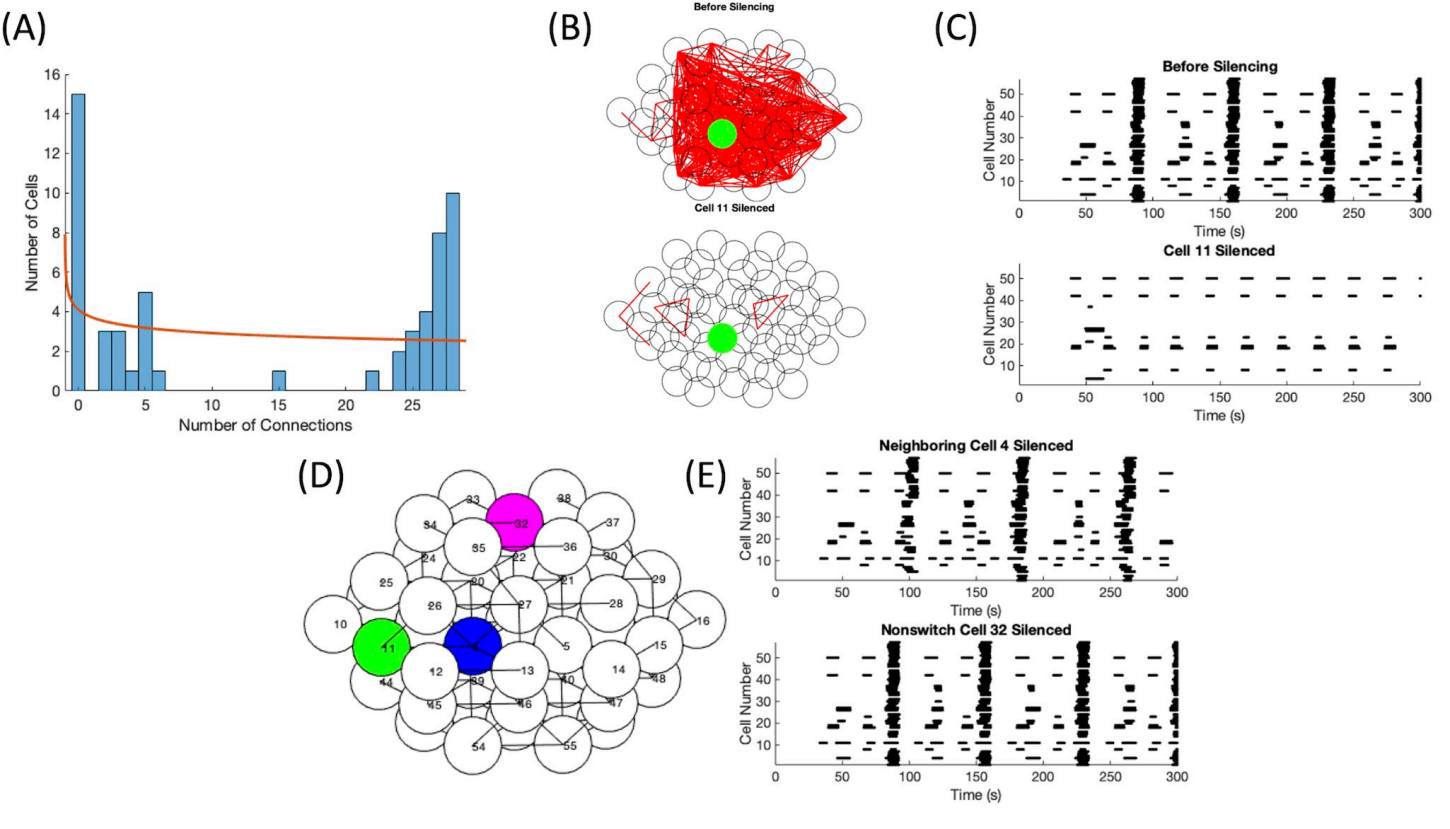
Switch cell silencing at weak coupling. This parameter set was drawn from *g*_*K*(*ATP*)_ ∼ *N*(145, 14.5) pS and *g*_*c*_ ∼ *N*(5, 2.5) pS. (A) The distribution of functional connections fit a power law with *R*^2^ = 0.028. (B) The functional connectivity graphs before (top panel) and after (bottom panel) the silencing of switch cell 11. (C) The raster plots before (top panel) and after (bottom panel) switch cell 11 was silenced. (D) The islet location of the switch cell 11 in green, neighboring cell 4 in blue, and randomly selected cell 32 in magenta. (E) The raster plots when neighboring cell 4 (top panel) and random cell 32 (bottom panel) were silenced.

**Fig 8:**
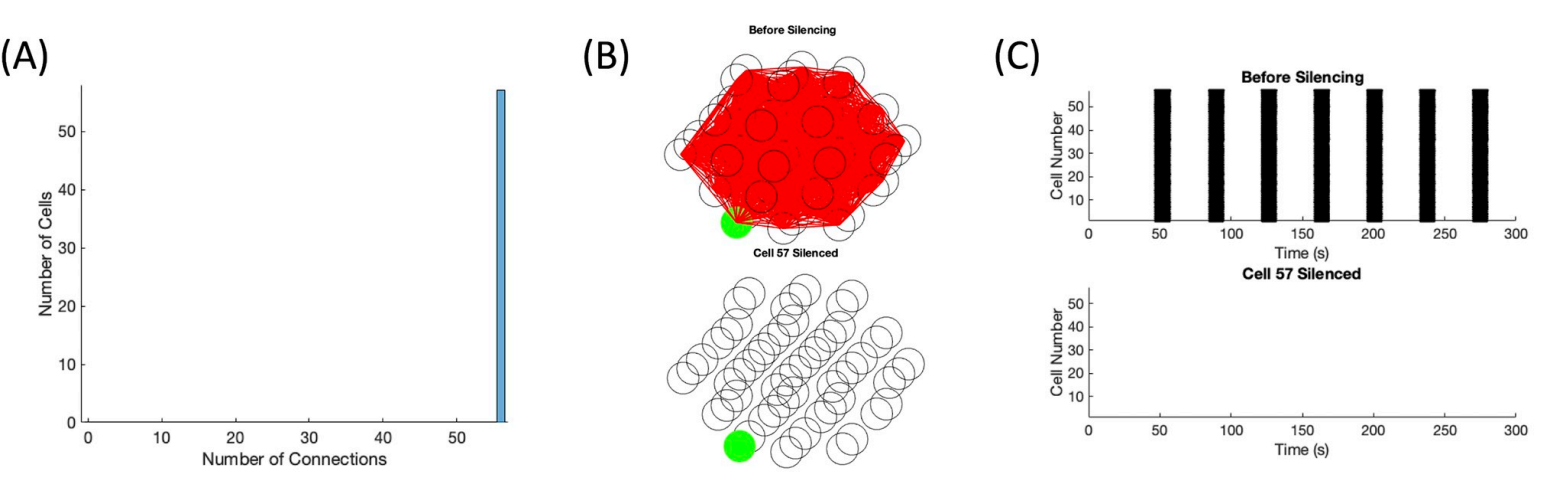
Switch cell silencing at strong coupling. This parameter set was drawn from *g*_*K*(*ATP*)_ ∼ *N*(135, 13.5) pS and *g*_*c*_ ∼ *N*(200, 100) pS. (A) No fit was found for the distribution of functional connections. (B) The functional connectivity graphs before (top panel) and after (bottom panel) the silencing of switch cell 57. (C) The raster plots before (top panel) and after (bottom panel) switch cell 57 was silenced.

Two examples of this drastic loss of activity can be observed in Figs 7 and 8. For Fig 7, the coupling and excitability was set lower with *g*_*c*_ ∼ *N*(5, 2.5) pS and *g*_*K*(*ATP*)_ ∼ *N*(145, 14.5) pS. When cell 11 was silenced, the majority of the islet became silent, indicating that cell 11 was a synchronization hub as seen in Fig 7(C). The calcium traces corresponding to Fig 7(C) can be found in S6 Fig in S2 Appendix. The functional network transitioned from highly connected before silencing to only nine functional connections, displayed in Fig 7(B). However, cell 11 was not functionally connected to any cell in the islet as its period is a fourth of the period of the majority of cells in the islet, and thus was not a functional hub. Fig 7(A) illustrated the poor power law fit for the functional connectivity with *R*^2^ = 0.028. Thus, the network was not classified as scale-free. In comparison, two control silencing simulations were tested: a neighboring cell of the switch cell and a random cell in the islet. The locations of the neighboring cell 4 (in blue) and the random cell 32 (in magenta) are shown relative to switch cell 11 (in green) in Fig 7(D). The silencing of neighboring cell 4 impacted the islet activity, seen in Fig 7(E) top panel, compared to the before silencing raster plot in Fig 7(C). However, the majority of cells in the islet continued to burst, indicating that cell 4 is not a switch cell. Meanwhile, there was a minimal difference between the islet behavior in the raster plot before silencing and with the silencing of cell 32, shown in Fig 7(E) bottom panel.

When *g*_*c*_ was increased to *g*_*c*_ ∼ *N*(200, 100) pS, cells had to be made more active in the islet to overcome the coupling to silent cells. Thus, the K(ATP) conductance values were selected from the distribution, *g*_*K*(*ATP*)_ ∼ *N*(135, 13.5) pS, for the example shown in Fig 8. The silencing of cell 57 led to a complete silencing of the islet, classifying it as a synchronization hub, and a total loss of functional connectivity, as seen in Fig 8(B) and 8(C), respectively. The calcium traces corresponding to Fig 8(C) can be found in S7 Fig in S2 Appendix. Due to the high structural connectivity, every cell was bursting together in the initial simulation, resulting in every cell functionally connected to each other. Therefore, the functional connectivity does not follow a power law distribution, illustrated by Fig 8(A). To differentiate these cells from the hub cells in the work of Johnston et al. [14], we have relabelled the synchronization hubs as switch cells, as these cells seem to act as a switch turning the activity of the islet on or off. Switch cells are synchronization hubs that are not functional hubs.

As *g*_*c*_ changes, the presence of switch cells is altered from no switch cells at all to the occurrence of different or multiple switch cells. For the parameter set with *g*_*c*_ ∼ *N*(5, 2.5) shown in Fig 7, the gap junction conductances were doubled. The resulting islet had two switch cells: cell 11 and cell 26. When the gap junctional conductance was increased by a factor of 5, the islet had 4 switch cells: cells 4, 11, 26, and 27. A comparison of the raster plots for each scaling factor is in the supplemental information (S8 Fig in S2 Appendix). The number of islets with a switch cell is biphasic in coupling strength with the number increasing from lower levels of coupling, peaking, then decreasing as coupling becomes even stronger (S3 Table in S1 Appendix). Furthermore, the number of switch cells per islet increases with coupling strength and excitability (S4 Table in S1 Appendix).

### Spatially distributed parameters can also lead to switch cells

The notion of cell parameter clustering, recently investigated [9], gave rise to the idea of spatially distributed parameters. After selecting cell parameters for islets as described in the Methods section, cells in the islet were systematically silenced to search for switch cells.

**Fig 9:**
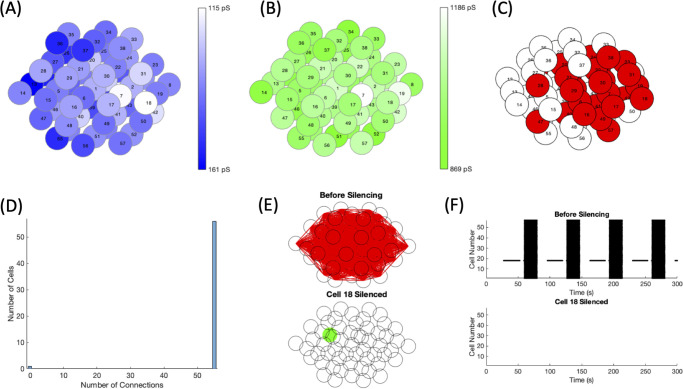
Switch cell silencing with spatially distributed cell parameters. The distribution of (A) *g*_*K*(*ATP*)_ and (B) *g*_*Ca*_ of the islet are denoted with the darker blue and darker green representing higher values. The orienting cell was selected as cell 7. (C) The intrinsically active cells in the islet are shown in red. (D) The distribution of functional connections did not have a fit. (E) The functional connectivity graphs before (top panel) and after (bottom panel) silencing switch cell 18. The raster plots before (top panel) and after (bottom panel) switch cell 18 was silenced.

Fig 9 depicts an example of a spatially distributed islet with switch cells with strong coupling (*g*_*c*_ ∼ *N*(200, 100)). In Fig 9(A) and 9(B), the distributions of *g*_*K*(*ATP*)_ and *g*_*Ca*_ are shown, respectively. The orienting cell was randomly chosen as cell 7. As cell 7 had parameters selected to be active and *g*_*K*(*ATP*)_ increases and *g*_*Ca*_ decreases radially further from cell 7, there is a cluster of intrinsically active cells surrounding cell 7 displayed in red in Fig 9(C). There were two switch cells uncovered in the islet, cells 18 and 19. The impact of silencing cell 18 is shown in Fig 9(E) and 9(F). The calcium traces corresponding to Fig 9(F) can be found in S9 Fig in S2 Appendix. The initial high connectivity in the top panel and high synchronization in the top raster plot gave way to no activity and thus no connectivity in the bottom panels when cell 18 is silenced. It is interesting to note that both switch cells were neighbors of the orienting cell 7, but the orienting cell 7 was not a switch cell itself. The cell with a differing period than the remaining islet in the top raster plot was the switch cell 18, which was highly active as its *g*_*K*(*ATP*)_ was low (115.28 pS). Due to its period, its trace was not highly correlated with any other cell in the network, leading to no functional connections for cell 18. However, every other cell was behaving similarly, leading to 56 cells with 55 functional connections and one cell with no functional connections. Fig 9(D) illustrates the lack of a power law fit for the functional connectivity. The silencing of cell 19 showed very similar behavior to the silencing of cell 18, with the only difference being the raster plot while silencing. As cell 18 was highly active, it continued to burst even when cell 19 was silenced.

### Switch cells can have a variety of properties

**Fig 10:**
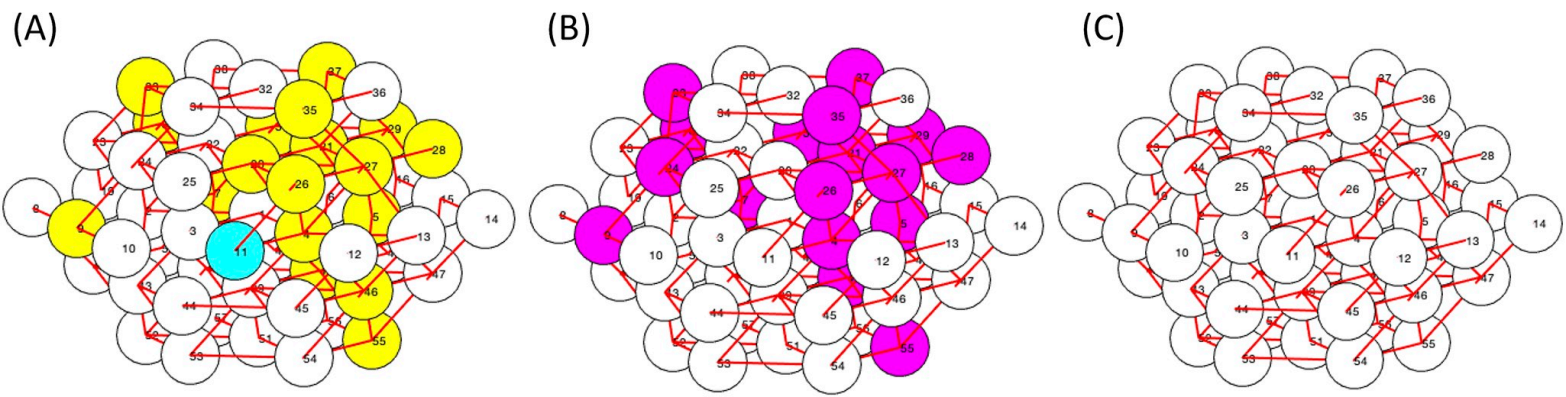
Cell parameter sensitivity for switch cell islets. Perturbing cell parameters (A) *g*_*Ca*_, (B) *g*_*K*(*ATP*)_, and (C) *g*_*K*_, *g*_*S*_, or *k*_*Ca*_ resulted in being pushed out of the loss of activity regime with both a 10% decrease and 10% increase (blue cell), with a 10% increase (yellow cells), or with a 10% decrease (magenta cells). The white cells represent cells with no observable change. The switch cell for this parameter set is cell 11. The red lines connecting cells denote the nonzero gap junction connections in the network.

A sensitivity analysis was performed on the cell parameters in the first switch cell example with lower coupling (in Fig 7), the results of which are shown in Fig 10. Systematically each cell parameter was increased or decreased by 10%, after which the simulations before and after silencing the switch cell were rerun. When increasing *g*_*Ca*_ for some cells, indicated in yellow in Fig 10(A), the silencing of the switch cell was no longer sufficient for the loss of islet-wide bursting. The excitability of the yellow cell was raised such that it was above the threshold for activity. This led to a cluster of active cells exhibiting bursting activity without the stimulus of the switch cell. Similarly, decreasing *g*_*K*(*ATP*)_ led to more excitable cells that pushed the islet out of the switch cell regime, seen in Fig 10(B) with the magenta cells. Perturbations to the cell parameters *g*_*K*_, *g*_*S*_, and *k*_*Ca*_ had a minimal to no effect on the behavior of the network regarding the existence of switch cell 11, observed in Fig 10(C), which is reasonable since these parameters do not strongly impact the excitability of the individual cells (i.e. do not exhibit a Hopf bifurcation nor other bifurcations that might lead to oscillations such as homoclinic or saddle node on an invariant circle). Interestingly, perturbing the *g*_*Ca*_ of the switch cell in either direction had a large impact on the islet behavior. When *g*_*Ca*_ was increased by 10%, the switch cell was spiking and unable to excite the rest of the islet. On the other hand, a 10% decrease in *g*_*Ca*_ of the switch cell lowered its excitability, resulting in a silent cell and a mainly silent islet.

**Fig 11:**
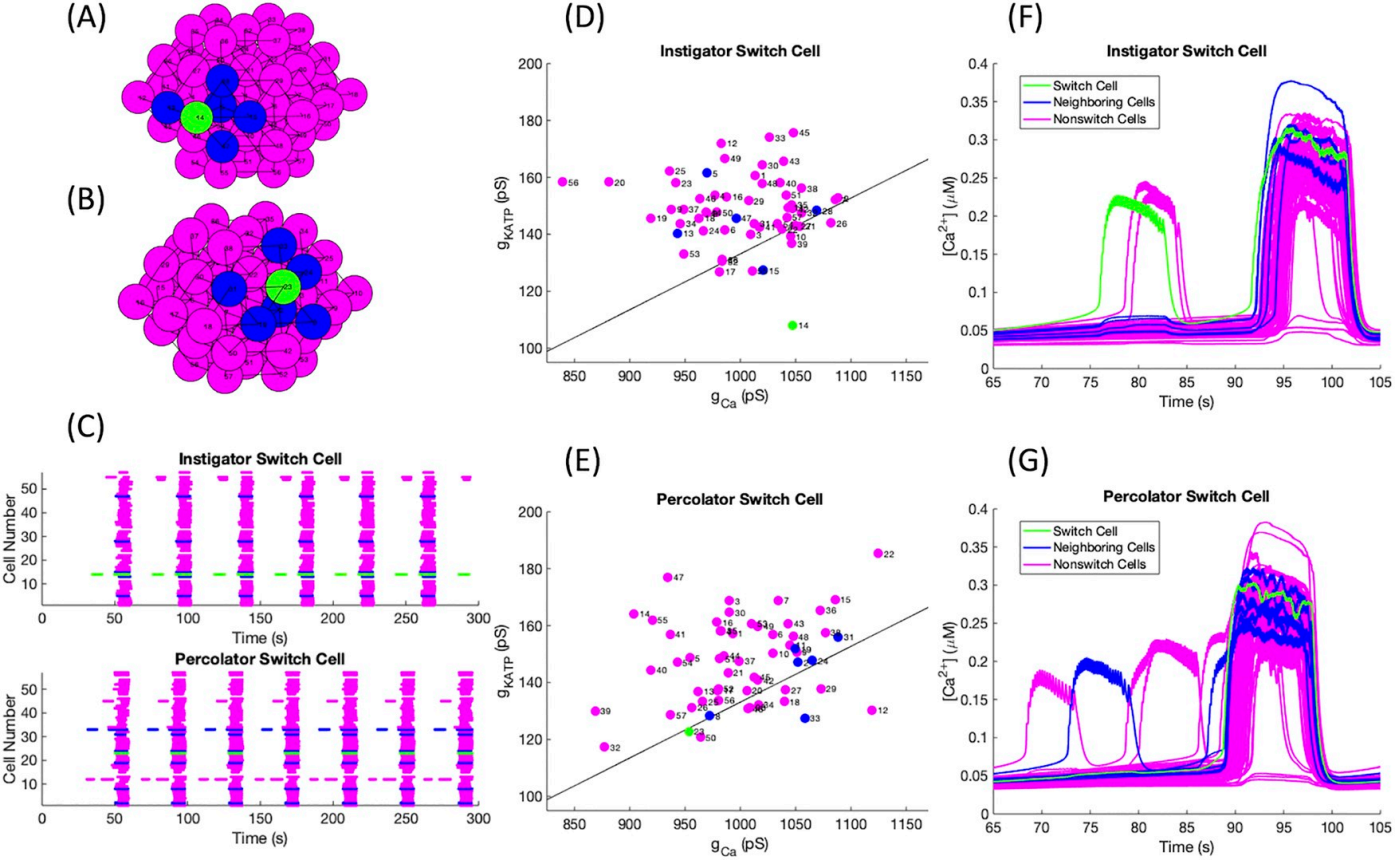
Initiator versus percolator switch cell. Switch cells are denoted in green, their gap junctionally connected neighbors in blue, and remaining cells in magenta. The islet structure with (A) an initiator and (B) a percolator switch cell. (C) Raster plots for islets with switch cells in green to indicate the differences in period between initiators (top panel) and percolators (bottom panel). The activation parameters for each cell in the islet are shown for a parameter set with (D) an initiator and (E) a percolator switch cell. The black line represents the Hopf bifurcation below which a cell, when isolated, will burst. Calcium time courses for (F) an initiator and (G) a percolator switch cell relative to the rest of the islet.

Switch cells can be classified into two categories: **initiators** that are at the start of the calcium wave spreading through the islet or **percolators** which are permissive in the propagation of calcium beyond nearest-neighbor local networks, but do not initiate the wave. The initiator switch cells have a low *g*_*K*(*ATP*)_ and a high *g*_*Ca*_, values that are separated in parameter space from those of the remaining cells in the islet as shown in the examples in Fig 11(D) compared to a parameter set with a percolator switch cell in Fig 11(E). The Hopf curve indicated by the black line in Fig 11(D) and 11(E) was calculated by using data points found in XPPAUT that were then fitted to a curve using MATLAB’s fit function. The resulting line shown in the figure had a goodness of fit of *R*^2^ = 0.98. The cells below the Hopf curve were active cells when isolated, while the cells above the curve were intrinsically silent. Percolator switch cells are not required to be active when isolated, but need to be close enough to threshold that a small stimulus from a neighboring cell can pull them into bursting activity. It is important for a percolator switch cell to be gap junctionally connected to a highly active cell, as seen in Fig 11(E) with switch cell 23 and cell 33. A notable difference between the two types of switch cells is their periods compared to the islet burst period shown in Fig 11(C). As initiator switch cells are more active, they burst at a higher frequency than most cells in the islet. Since the action of a percolator is to permit the spreading of a calcium wave, it only bursts when the majority of the network does. Thus, percolators will have a large number of functional connections while the initiator will have very few to none. However, neither type of switch cell is a hub cell in the sense of high functional or structural connectivity. The initiator cells are similar to the leader cells introduced in [19]. The initiators are the first cells to respond in a situation similar to stimulatory glucose, which then spreads electrically though gap junctional coupling to neighboring cells, seen in Fig 11(F). However, without the presence of the initiator cell, the electrical wave was not observed, supporting the notion that they start the wave of activity within the islet. Meanwhile percolators have a slightly delayed response, but still turn on near the beginning of a calcium wave after one or more of their neighbors, as shown in Fig 11(G). When the percolator is silenced, only the active nearest neighbors of the percolator will burst, as the wave is unable to spread to the rest of the islet. We have attempted to gain a more quantitative understanding behind the two types of switch cells, but have so far not been able to determine their exact characteristics.

**Fig 12:**
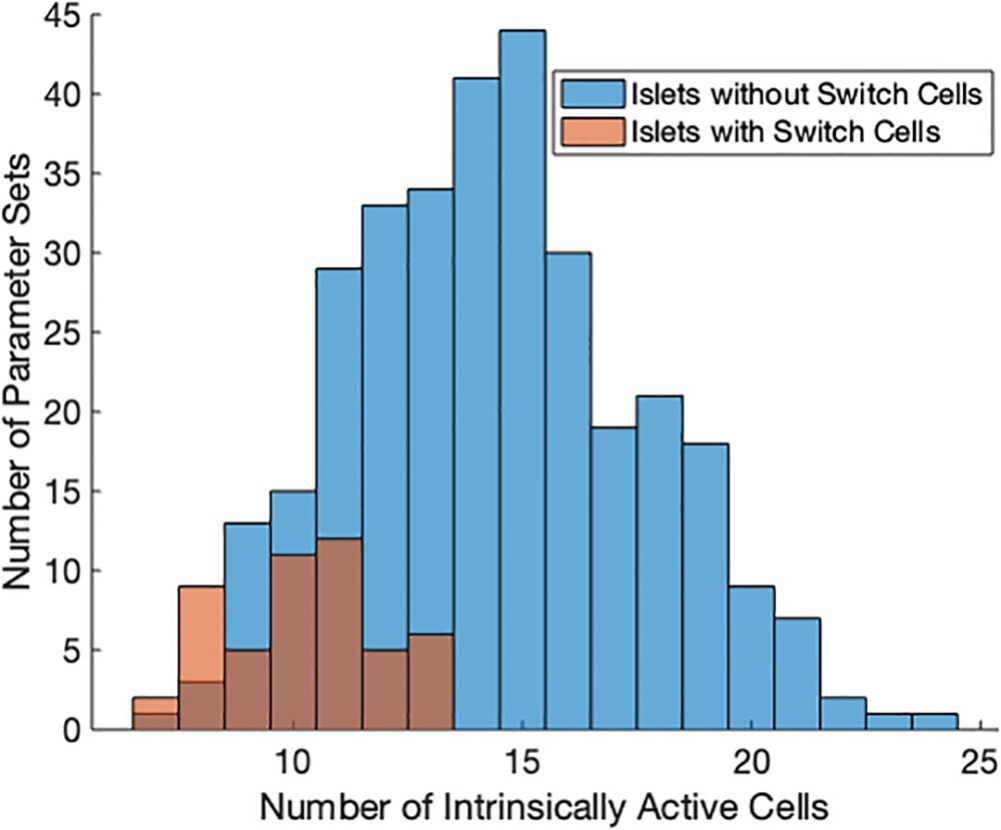
Histogram of active cells. The number of active cells per parameter set for parameter sets with switch cells (red, N = 50) and some of the parameter sets without switch cells (blue, N = 321). The region of overlap is denoted in the darker brown color. The distributions for parameters are *g*_*Ca*_ ∼ *N*(1000, 50) and *g*_*K*(*ATP*)_ ∼ *N*(145, 14.5) where *g*_*c*_ ∼ *N*(5, 2.5).

With the distributions *g*_*Ca*_ ∼ *N*(1000, 50) and *g*_*K*(*ATP*)_ ∼ *N*(145, 14.5), the probability of an individual cell being intrinsically active was 24.83%. Thus the expected number of cells that were active when uncoupled in this system was 14.15 with standard deviation 3.26. It is notable that all parameter sets found with at least one switch cells had 13 or fewer active cells as seen in Fig 12. In order for a cell to behave as a switch cell, the number of intrinsically active cells in the rest of the islet needed to be smaller than average, or silencing the switch cell would not silence most of the islet. For this excitability level and *g*_*c*_ ∼ *N*(5, 2.5), it was found that the average number of intrinsically active cells for parameter sets with switch cells was 10.21 ± 1.7. However, many cells that were intrinsically active were held silent in the islet by gap junctional coupling to intrinsically silent cells. A small stimulus from a calcium wave started by an initiator switch cell activated these follower cells, spreading the wave to neighboring cells and leading to an islet-wide burst. If too many cells in the islet were intrinsically active, the network activity was not silenced with the silencing of any cell.

**Fig 13:**
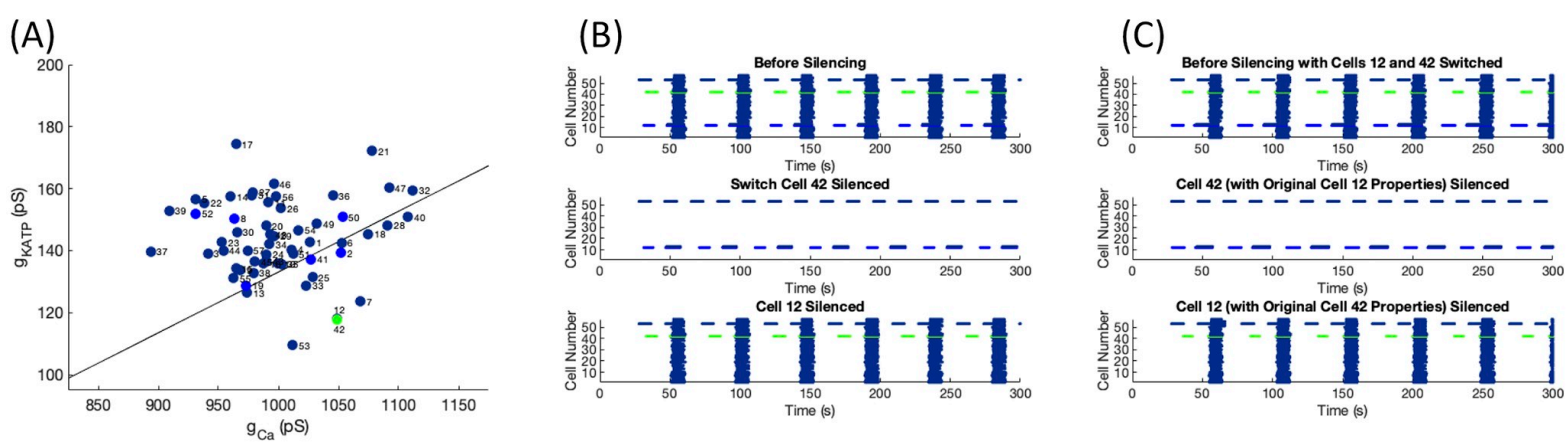
Swapping switch cell properties. (A) Activation parameters for cells in the islet with the switch cell in green, its neighbors in blue, and all other cells in navy. (B) Raster plots for the islet before silencing (top panel), with switch cell 42 silenced (middle panel), and for cell 12 silenced (bottom panel). (C) Raster plots for an islet with the cellular properties of cells 42 and 12 swapped before silencing (top panel), with the new cell 42 with properties of the original cell 12 silenced (middle panel), and for the new cell 12 with properties of the original cell 42 silenced (bottom panel).

Thus, while the excitability properties of the switch cells are critical, the connectivity properties of the network and cellular properties of the neighbors may be similarly important. An example of this point is shown in Fig 13. In this islet, cell 12 had very similar activation parameters to the switch cell 42. Both cells had a higher frequency of bursting than the islet-wide burst, shown in the top panel of Fig 13(B). However, islet-wide bursts continued when cell 12 was silenced shown in Fig 13(B) bottom panel and islet-wide bursting was lost when switch cell 42 was silenced in the middle panel. Fig 13(C) illustrates the minimal impact of swapping the cell parameters for cells 12 and 42. Cell 42 remained the switch cell while silencing cell 12 was unable to silence the islet, implying that the structural properties around switch cell 42 led to the loss of activity behavior.

## Discussion

Islets are well-coupled populations of heterogeneous cells, which have been often modeled as homogeneous due to the synchronizing effects of electrical coupling. Recent experiments have suggested that individual *β* cell heterogeneity can in some cases dominate the collective islet.

This study was a search for model islets of *β* cells that satisfied the conditions proposed experimentally for silencing an islet through individual hub cells: a scale-free functional connectivity network and a loss of connectivity and desynchronization with hub cell silencing [14]. While scale-free functional networks were found assuming heterogeneity of cell parameters and coupling, confirming previous computational work [25], and these networks lost functional connectivity when the functional hub cell was silenced, desynchronization was never observed. As this approach proved unsuccessful, predetermination of hub cells was tested, similar to previous work [17]. For desynchronization with silencing, it was necessary for the predetermined “hub” cell to be the only intrinsically active cell in the islet. However, it has been shown experimentally that most cells can oscillate independent of the activity of neighboring cells [27]. These islets were also not characterized as scale-free nor were the predetermined “hub” cells considered to be functional hubs cells, bringing into question the utility of functional connectivity measure.

Multiple methods of determining functional connectivity have been utilized on pancreatic islets. The more straightforward Pearson correlations have shown islets as small world networks [25, 28, 32, 33]. The binarized method, used in this study, has revealed scale-free properties in the islets of zebrafish, mice, and humans [14, 19]. In a comparison between the two methods, a noticeable difference is the connectivity of silent cells. The calcium traces of two silent cells are highly correlated and thus functionally connected by the Pearson correlation method. As the binarized method puts an emphasis on the activity of cells in functional connections, two silent cells are not considered functionally connected. In mouse islets, the binarized method led to scale-free networks while the Pearson correlation method did not [19].

The binarized method of functional connectivity was unsuccessful in assisting in the uncovering of hub cells that silence the islet. Since it focuses on the correlations in activity, using the degree centrality on the functional connectivity network results in locating the cells with the most common behavior in the islet. By considering this cell the leader of islet behavior, the assumption is being made that correlation implies causation. However, silencing the functional hub cell was unable to desynchronize the islet in our simulations, dispelling this notion. A trade off was found between scale-free connectivity, which is necessary for functional hub cells, and islet desynchronization. Highly functionally connected cells, which have many strong gap junctional connections, are more likely to have their activity silenced than initiate a wave. On the other hand, a less connected cell is more likely to remain active and initiate a smaller local wave that may spread through out the islet due to connectivity of neighboring cells.

In order to find cells that would replicate the desynchronization of an islet with individual cell silencing, cells were systematically silenced computationally, searching for a dissipation of coordinated oscillations. Depending on the conductance levels, 15-65% of active islets tested contained at least one switch cell, which led to a loss of islet-wide bursting when silenced. Two types of switch cells were found: initiator and percolator. While highly active cellular parameters were needed for an initiator switch cell, the structural properties and cellular properties of neighbors also played a critical role in the emergence of switch cells.

A motivation behind silencing a cell is to study the impact of the loss of that cell’s activity on the network, imitating the effect on the islet of that cell becoming dysfunctional or dying. However, silencing a cell by hyperpolarizing it may have some unintended consequences. When the genetically added pumps are activated optically, the membrane potential of the targeted cell decreases due to the chloride ions moving into the cell. This hyperpolarization can lead to the hyperpolarization of neighboring cells through gap junctions, which can cause an islet to go silent, especially a strongly connected islet. The number of switch cells with differing behavior between isolation and silencing grew as the coupling was made stronger, implying that the silencing technique may not be suitable to determine the effect of cell removal from the network if the islet is tightly coupled. While experimentally switch cells have not been found, the process to find them is difficult currently as each cell would need to be silenced in order to measure the effect on the remaining cells in the islet. With further computational work, characteristics of switch cells can be determined, assisting locating these cells in experiments at intermediate levels of glucose.

As the network properties were the focus, a simple model looking only at the electrical and calcium dynamics was selected. The metabolic impact was included through the conductance of the ATP/ADP ratio dependent potassium channel (*g*_*K*(*ATP*)_), which was held constant for a given cell. However, that ratio and thus the conductance is dynamic due to the phosphorylation of ADP through metabolism and the dephosphorylation of ATP for energy use. Our results can be interpreted as the activity of an islet at a certain level of glucose. A given islet may have a low probability of having switch cells for a certain glucose level and may not consistently have the same switch cells, but different switch cells may emerge at differing levels of glucose, leading to a possible dysfunction in behavior at specific glucose levels. Johnston et al. had shown that the hub cells had an increased expression of SERCA protein [14]. While the model used for this study did not have a direct parameter representing the SERCA pumps, the *k*_*Ca*_ parameter, which denotes a general calcium pump, was taken to be heterogeneous to account for differing levels of protein expression.

However, the scale-free, hub, and switch experiments were repeated for a more sophisticated model [34] containing both a dynamic metabolic component and a direct parameter for the SERCA pumps. While certain islets were shown to be scale-free, the silencing of the functional hub cell was unable to desynchronize the islet, supporting the contradiction between scale-free networks and desynchronization. Meanwhile, with a parameter regime consistent with that used in this study, islets with the more complex model were shown to have switch cells that are able to silence the activity of the islet. Further work with this model is needed to determine the cellular and network properties required for the emergence of switch cells. This work also focused on small networks with 57 *β* cells for efficiency in testing many cases exhaustively. Even though synchronization was achieved, it is important to note that smaller islets may be less likely to synchronize in reality. Thus, it is critical that these experiments are repeated on islets of a larger size. While similar results have been found in a few trial cases, more work will need to be done.

In a recent discussion on the electrophysiological basis of hub cells [35–37], there was debate on the ability for a hub cell to silence an entire islet. Our results suggest that such an effect on an islet’s electrical activity is indeed possible under certain specific conditions. Understanding these conditions more fully using mathematical modeling may lead to resolving, or at least leading to a resolution, of the current controversy surrounding hub cells.

In summary, islets were previously thought to be quite democratic in nature where all cells contributed more or less equally to islet behavior. Recent experiments [14, 19] and computational results [17] have introduced the idea of hub cells that govern islet activity. While the experimental studies [14, 19] demonstrated that the hub cells were highly functionally connected, the simulation results of [17] argue that scale-free properties are not required for desynchronization with hub cell silencing, only partially explaining the experimental data [14, 19]. In this paper, we have identified a different type of cell, switch cell, whose loss has a similar effect of silencing the islet, that may be able to offer a different explanation behind the experimental data [35–37]. While switch cells do not relate to functional connectivity, some switch cells can be leader cells [9, 19]. Gaining further insight into the mechanisms behind switch cells may assist in understanding the hub cell experiments and the loss of synchronization occurring in pathological conditions such as type 2 diabetes.

## Supporting information

S1 Appendix Text and tables. (PDF)

S2 Appendix Additional figures. (PDF)

## References

[1] Cabrera O, Berman DM, Kenyon NS, Ricordi C, Berggren PO, Caicedo A. The unique cytoarchitecture of human pancreatic islets has implications for islet cell function. Proceedings of the National Academy of Sciences. 2006;103(7):2334–2339. 10.1073/pnas.0510790103 PMC1413730 16461897

[2] Weyer C, Bogardus C, Mott DM, Pratley RE. The natural history of insulin secretory dysfunction and insulin resistance in the pathogenesis of type 2 diabetes mellitus. The Journal of Clinical Investigation. 1999;104(6):787–794. 10.1172/JCI7231 10491414 PMC408438

[3] Rorsman P, Braun M. Regulation of insulin secretion in human pancreatic islets. Annual Review of Physiology. 2013;75:155–179. 10.1146/annurev-physiol-030212-183754 22974438

[4] Gilon P, Henquin JC. Influence of membrane potential changes on cytoplasmic Ca^2+^ concentration in an electrically excitable cell, the insulin-secreting pancreatic B-cell. Journal of Biological Chemistry. 1992;267(29):20713–20720. 10.1016/S0021-9258(19)36744-4 1400388

[5] Eddlestone GT, Gon calves A, Bangham JA, Rojas E. Electrical coupling between cells in islets of Langerhans from mouse. Journal of Membrane Biology. 1984;77(1):1–14. 10.1007/BF01871095 6321740

[6] Ravier MA, Güldenagel M, Charollais A, Gjinovci A, Caille D, Söhl G, et al. Loss of connexin36 channels alters *β*-cell coupling, islet synchronization of glucose-induced Ca^2+^ and insulin oscillations, and basal insulin release. Diabetes. 2005;54(6):1798–1807. 10.2337/diabetes.54.6.1798 15919802

[7] Head WS, Orseth ML, Nunemaker CS, Satin LS, Piston DW, Benninger RK. Connexin-36 gap junctions regulate in vivo first-and second-phase insulin secretion dynamics and glucose tolerance in the conscious mouse. Diabetes. 2012;61(7):1700–1707. 10.2337/db11-1312 22511206 PMC3379660

[8] Benninger RK, Zhang M, Head WS, Satin LS, Piston DW. Gap junction coupling and calcium waves in the pancreatic islet. Biophysical Journal. 2008;95(11):5048–5061. 10.1529/biophysj.108.140863 18805925 PMC2586567

[9] Westacott MJ, Ludin NW, Benninger RK. Spatially organized *β*-cell subpopulations control electrical dynamics across islets of Langerhans. Biophysical Journal. 2017;113(5):1093–1108. 10.1016/j.bpj.2017.07.021 28877492 PMC5658715

[10] Benninger RK, Hodson DJ. New understanding of *β*-cell heterogeneity and in situ islet function. Diabetes. 2018;67(4):537–547. 10.2337/dbi17-0040 29559510 PMC5860861

[11] O’Rahilly S, Turner RC, Matthews DR. Impaired pulsatile secretion of insulin in relatives of patients with non-insulin-dependent diabetes. New England Journal of Medicine. 1988;318(19):1225–1230. 10.1056/NEJM198805123181902 3283553

[12] Matthews D, Lang D, Burnett M, Turner R. Control of pulsatile insulin secretion in man. Diabetologia. 1983;24(4):231–237. 10.1007/BF00282705 6345247

[13] Polonsky KS, Given BD, Hirsch LJ, Tillil H, Shapiro ET, Beebe C, et al. Abnormal patterns of insulin secretion in non-insulin-dependent diabetes mellitus. New England Journal of Medicine. 1988;318(19):1231–1239. 10.1056/NEJM198805123181903 3283554

[14] Johnston NR, Mitchell RK, Haythorne E, Pessoa MP, Semplici F, Ferrer J, et al. Beta cell hubs dictate pancreatic islet responses to glucose. Cell Metabolism. 2016;24(3):389–401. 10.1016/j.cmet.2016.06.020 27452146 PMC5031557

[15] Rubinov M, Sporns O. Complex network measures of brain connectivity: Uses and interpretations. Neuroimage. 2010;52(3):1059–1069. 10.1016/j.neuroimage.2009.10.003 19819337

[16] Rutter GA, Hodson DJ. Beta cell connectivity in pancreatic islets: A type 2 diabetes target? Cellular and Molecular Life Sciences. 2015;72(3):453–467. 10.1007/s00018-014-1755-4 25323131 PMC11113448

[17] Lei CL, Kellard JA, Hara M, Johnson JD, Rodriguez B, Briant LJ. Beta-cell hubs maintain Ca^2+^ oscillations in human and mouse islet simulations. Islets. 2018;10(4):151–167. 10.1080/19382014.2018.1493316 30142036 PMC6113907

[18] Loppini A, Chiodo L. Biophysical modeling of *β*-cells networks: Realistic architectures and heterogeneity effects. Biophysical Chemistry. 2019; p. 106247. 10.1016/j.bpc.2019.106247 31472460

[19] Salem V, Silva LD, Suba K, Georgiadou E, Gharavy SNM, Akhtar N, et al. Leader *β*-cells coordinate Ca^2+^ dynamics across pancreatic islets in vivo. Nature Metabolism. 2019;1(6):615. 10.1038/s42255-019-0075-2 32694805 PMC7617060

[20] Granger CW. Investigating causal relations by econometric models and cross-spectral methods. Econometrica: Journal of the Econometric Society. 1969; p. 424–438.

[21] Sherman A. Contributions of modeling to understanding stimulus-secretion coupling in pancreatic beta-cells. American Journal of Physiology-Endocrinology And Metabolism. 1996;271(2):E362–E372. 10.1152/ajpendo.1996.271.2.E362 8770032

[22] Benninger RK, Hutchens T, Head WS, McCaughey MJ, Zhang M, Le Marchand SJ, et al. Intrinsic islet heterogeneity and gap junction coupling determine spatiotemporal Ca^2+^ wave dynamics. Biophysical Journal. 2014;107(11):2723–2733. 10.1016/j.bpj.2014.10.048 25468351 PMC4255172

[23] Gutierrez GD, Gromada J, Sussel L. Heterogeneity of the pancreatic beta cell. Frontiers in Genetics. 2017;8:22. 10.3389/fgene.2017.00022 28321233 PMC5337801

[24] Xavier GDS, Rutter GA. Metabolic and functional heterogeneity in pancreatic *β* cells. Journal of Molecular Biology. 2019;. 10.1016/j.jmb.2019.08.005 31419404

[25] Cappon G, Pedersen MG. Heterogeneity and nearest-neighbor coupling can explain small-worldness and wave properties in pancreatic islets. Chaos: An Interdisciplinary Journal of Nonlinear Science. 2016;26(5):531031–531037. 10.1063/1.4949020 27249943

[26] Nittala A, Ghosh S, Wang X. Investigating the role of islet cytoarchitecture in its oscillation using a new *β*-cell cluster model. PLoS One. 2007;2(10):e983. 10.1371/journal.pone.0000983 17912360 PMC1991600

[27] Scarl RT, Corbin KL, Vann NW, Smith HM, Satin LS, Sherman A, et al. Intact pancreatic islets and dispersed beta-cells both generate intracellular calcium oscillations but differ in their responsiveness to glucose. Cell Calcium. 2019;83:102081. 10.1016/j.ceca.2019.102081 31563790 PMC6983470

[28] Stožer A, Gosak M, Dolenšek J, Perc M, Marhl M, Rupnik MS, et al. Functional connectivity in islets of Langerhans from mouse pancreas tissue slices. PLoS Computational Biology. 2013;9(2):e1002923. 10.1371/journal.pcbi.1002923 23468610 PMC3585390

[29] Hodson D, Molino F, Fontanaud P, Bonnefont X, Mollard P. Investigating and modelling pituitary endocrine network function. Journal of Neuroendocrinology. 2010;22(12):1217–1225. 10.1111/j.1365-2826.2010.02052.x 20673299

[30] Barabási AL, Albert R. Emergence of scaling in random networks. Science. 1999;286(5439):509–512. 10.1126/science.286.5439.509 10521342

[31] Barabasi AL, Oltvai ZN. Network biology: Understanding the cell’s functional organization. Nature Reviews Genetics. 2004;5(2):101–113. 10.1038/nrg1272 14735121

[32] Markovič R, Stožer A, Gosak M, Dolenšek J, Marhl M, Rupnik MS. Progressive glucose stimulation of islet beta cells reveals a transition from segregated to integrated modular functional connectivity patterns. Scientific Reports. 2015;5:7845. 10.1038/srep07845 25598507 PMC4297961

[33] Barua AK, Goel P. Isles within islets: The lattice origin of small-world networks in pancreatic tissues. Physica D: Nonlinear Phenomena. 2016;315:49–57. 10.1016/j.physd.2015.07.009

[34] Marinelli I, Vo T, Gerardo-Giorda L, Bertram R. Transitions between bursting modes in the integrated oscillator model for pancreatic *β*-cells. Journal of Theoretical Biology. 2018;454:310–319. 10.1016/j.jtbi.2018.06.017 29935201

[35] Satin LS, Zhang Q, Rorsman P. “Take Me To Your Leader”: An Electrophysiological Appraisal of the Role of Hub Cells in Pancreatic Islets. Diabetes. 2020;69(5):830–836. 10.2337/dbi19-0012 32312899 PMC7171959

[36] Rutter GA, Ninov N, Salem V, Hodson DJ. Comment on Satin et al. “Take Me To Your Leader”: An Electrophysiological Appraisal of the Role of Hub Cells in Pancreatic Islets. Diabetes 2020;69:830–836. Diabetes. 2020;69(9):e10–e11. 10.2337/db20-0501 32820056 PMC7458040

[37] Satin LS, Rorsman P. Response to Comment on Satin et al. “Take Me To Your Leader”: An Electrophysiological Appraisal of the Role of Hub Cells in Pancreatic Islets. Diabetes 2020;69:830–836. Diabetes. 2020;69(9):e12–e13. 10.2337/dbi20-0027 32312899 PMC7171959

