# Supplementary material for: Flipping the switch on the hub cell: Islet desynchronization through cell silencing
Source: PLoS One. 2021 Apr 8;16(4):e0248974. doi: 10.1371/journal.pone.0248974 (PMC8031451; doi:10.1371/journal.pone.0248974)
Supplement: S1 Appendix — (PDF) [file pone.0248974.s001.pdf]

S1 Appendix - Text and Tables

S1 Text - Model Equations A four variable model adapted from [1] was used for the simulations in this paper. The first variable tracked the membrane potential of a cell i with the dynamics given by the Hodgkin-Huxley like differential equation

$$-C_M \cdot \frac{dV_i}{dt} = I_{Ca}(V_i) + I_{K(ATP)}(V_i) + I_K(V_i, n_i) + I_S(V_i, s_i) + I_{Coup}(V_i) + I_{Cl}(V_i). \tag{1}$$

The membrane capacitance is given by C_M, listed in Table S1. I_{Ca} is the voltage dependent calcium current, $I_{K(ATP)}$ is the ATP/ADP ratio dependent potassium current, I_K is the delayed rectifier potassium current, I_S is the slow inhibitory potassium current, I_{Coup} is the gap junctional coupling current, and I_{Cl} is the optogenetically controlled chloride current. Their forms are given below:

$$I_{K(ATP)}(V_i) = g_{K(ATP)} \cdot (V_i - V_K) \tag{2}$$
$$I_{Ca}(V_i) = g_{Ca} \cdot m_\infty(V_i) \cdot (V_i - V_{Ca}) \tag{3}$$
$$I_K(V_i, n_i) = g_K \cdot n_i \cdot (V_i - V_K) \tag{4}$$
$$I_S(V_i, s_i) = g_S \cdot s_i \cdot (V_i - V_K) \tag{5}$$
$$I_{Coup}(V_i) = \sum_{\forall j \in N(i)} g_{c_{ij}} \cdot (V_i - V_j) \tag{6}$$
$$I_{Cl}(V_i) = g_{Cl} \cdot \sigma \cdot (V_i - V_{Cl}). \tag{7}$$

Each g_X represents the maximal conductances of the respective current and V_X denotes the ion's Nernst potential, listed in Table S1 and S2. The currents given in Equations S4 & S5 each have gating variables, n and s respectively, whose dynamics are given by the differential equations

$$\frac{dn_i}{dt} = \frac{n_\infty(V_i) - n_i}{\tau_n} \tag{8}$$
$$\frac{ds_i}{dt} = \frac{s_\infty(V_i) - s_i}{\tau_s}. \tag{9}$$

The time constants are represented by τ_n and τ_s, listed in Table S1, while the steady state forms of n and s are given by n_∞ and s_∞ respectively. As the gating variable for the voltage gated calcium channel reaches its equilibrium at a faster time scale, it is set to its steady state m_∞ in Equation S3. The functional forms for the steady states are

$$m_\infty(V_i) = \frac{1}{1 + \exp\left(\frac{V_i - V_m}{\theta_m}\right)} \tag{10}$$
$$n_\infty(V_i) = \frac{1}{1 + \exp\left(\frac{V_i - V_n}{\theta_n}\right)} \tag{11}$$
$$s_\infty(V_i) = \frac{1}{1 + \exp\left(\frac{V_i - V_s}{\theta_s}\right)}. \tag{12}$$

The parameters V_m, V_n, V_s, θ_m, θ_n, and θ_s are listed in Table S1. In Equation S6, the coupling current is summed over all neighbors of cell i, $N(i)$. The σ in Equation S7 denotes the optogenetic control over the halorhodopsin pumps in the membrane of cell i. When the chloride pumps are optogenetically activated, σ is set to 1 and otherwise is 0.

The last equation in the model denotes the change in calcium concentration

$$\frac{dc_i}{dt} = f \cdot [-\alpha \cdot I_{Ca}(V_i) - k_{Ca} \cdot c_i]. \tag{13}$$

The parameter f accounts for the fraction of free calcium, α converts the current to a rate of increase in the concentration, and k_{Ca} represents the rate at which calcium is being pumped out of the cell. Their values are given in Table S1 and S2.

S1 Table

Parameter	Symbol	Value
Membrane capacitance	C_M	5.3 pF
Optogentic chloride pump conductance	g_{Cl}	100 pS
Optogenetic control of the chloride pumps	σ	0 or 1
Potassium Nernst potential	V_K	-75 mV
Calcium Nernst potential	V_{Ca}	25 mV
Chloride Nernst potential	V_{Cl}	-70 mV
Half-maximal potential for m_∞	V_m	-20 mV
Half-maximal potential for n_∞	V_n	-17 mV
Half-maximal potential for s_∞	V_s	-22 mV
Slope of m_∞ at $V = V_m$	s_m	12 mV
Slope of n_∞ at $V = V_n$	s_n	5.6 mV
Slope of s_∞ at $V = V_s$	s_s	8 mV
Mean opening time of the slow inhibitory potassium channel	τ_s	20 s
Mean opening time of the voltage-gated potassium channel	τ_n	0.02 s
Fraction of free calcium in the intracellular space	f	0.01
Conversion factor from electrical gradient to chemical gradient	α	0.0045 μM/(fA $\times$ s)

S1 Table: Homogeneous Model Parameters. This assumes a typical cell surface area of 10^{-6}cm^2 (e.g. 5.3 μF/cm$^2 \rightarrow$ 5.3 pF and 100 μS/cm$^2 \rightarrow$ 100 pS)

S2 Table

Parameter or Initial Value	Symbol	Mean	SD
Voltage-gated potassium channel conductance	g_K	2700 pS	5%
Voltage-gated calcium channel conductance	g_{Ca}	1000 pS	5%
ATP/ADP dependent potassium channel conductance	$g_{K(ATP)}$	100-145 pS	5-10%
Slow inhibitory potassium channel conductance	g_S	200 pS	5%
Gap junction conductance	g_c	4-200 pS	50%
Calcium pump rate	k_{Ca}	0.2 s^{-1}	5%
Initial value for the membrane potential	V_0	-65 mV	5%
Initial value for the voltage gated potassium channel gating variable	n_0	0.5	5%
Initial value for the slow inhibitory potassium channel gating variable	s_0	1.01	5%
Initial value for the calcium concentration	c_0	0.05 μM	5%

S2 Table: Heterogeneous Model Parameters. Heterogeneous parameter and initial values were selected from distributions with the given means and standard deviations.

S3 Table

		g_c (pS)					
		5	10	25	50	100	200
$g_{K(ATP)}$ (pS)	135	0	0	4	30	27	17
	140	0	2	32	25	6	0
	143	1	9	27	13	2	0

S3 Table: The number of parameter sets with at least one switch cell out of 100 total parameter sets following distributions with the given mean values for $g_{K(ATP)}$ and g_c.

S4 Table

		g_c (pS)					
		5	10	25	50	100	200
$g_{K(ATP)}$ (pS)	135	N/A	N/A	7.5	13.8	31.963	50.882
	140	N/A	1	5.865	15	27.833	N/A
	143	2	4	9.189	6.615	2	N/A

S4 Table: The average number of switch cells per islet corresponding to Table S3. There are no switch islets over which to average in the N/A cases.

References

[1] Arthur Sherman. Contributions of modeling to understanding stimulus-secretion coupling in pancreatic beta-cells. *American Journal of Physiology-Endocrinology And Metabolism*, 271(2):E362–E372, 1996. PMID: 8770032.
