# Supplementary material for: Flipping the switch on the hub cell: Islet desynchronization through cell silencing
Source: PLoS One. 2021 Apr 8;16(4):e0248974. doi: 10.1371/journal.pone.0248974 (PMC8031451; doi:10.1371/journal.pone.0248974)
Supplement: S2 Appendix — (PDF) [file pone.0248974.s002.pdf]

S2 Appendix - Additional Figures

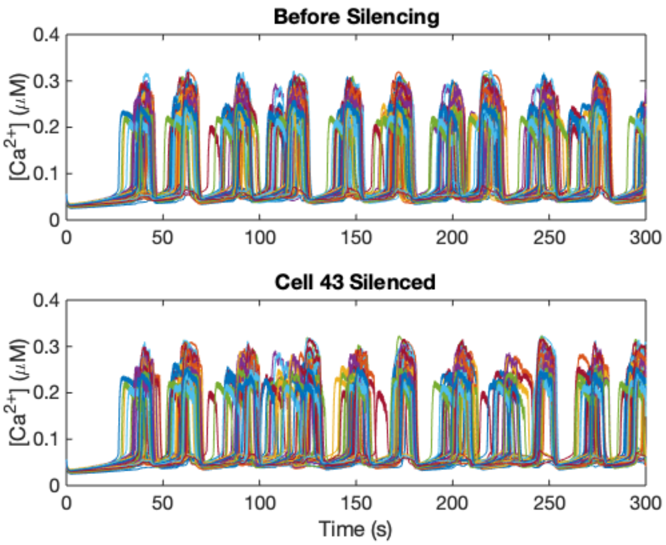

S1 Fig: The calcium traces for the pre-silencing (top) and silencing (bottom) simulations corresponding to Fig 4 (C) and (D) respectively, where this islet had the parameter distributions $g_{K(ATP)} \sim N(143, 14.3)$ pS and $g_c \sim N(4, 2)$ pS and was found to be scale-free.

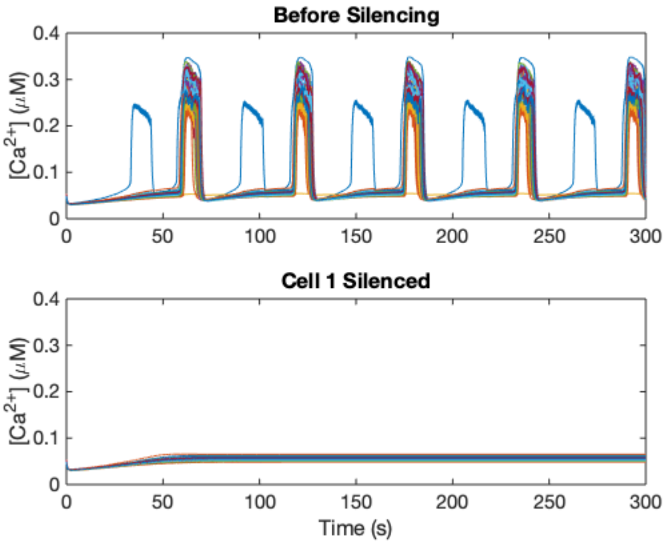

S2 Fig: The calcium traces for the pre-silencing (top) and silencing (bottom) simulations corresponding to Fig 5(C), where this islet had a predetermined "hub" cell.

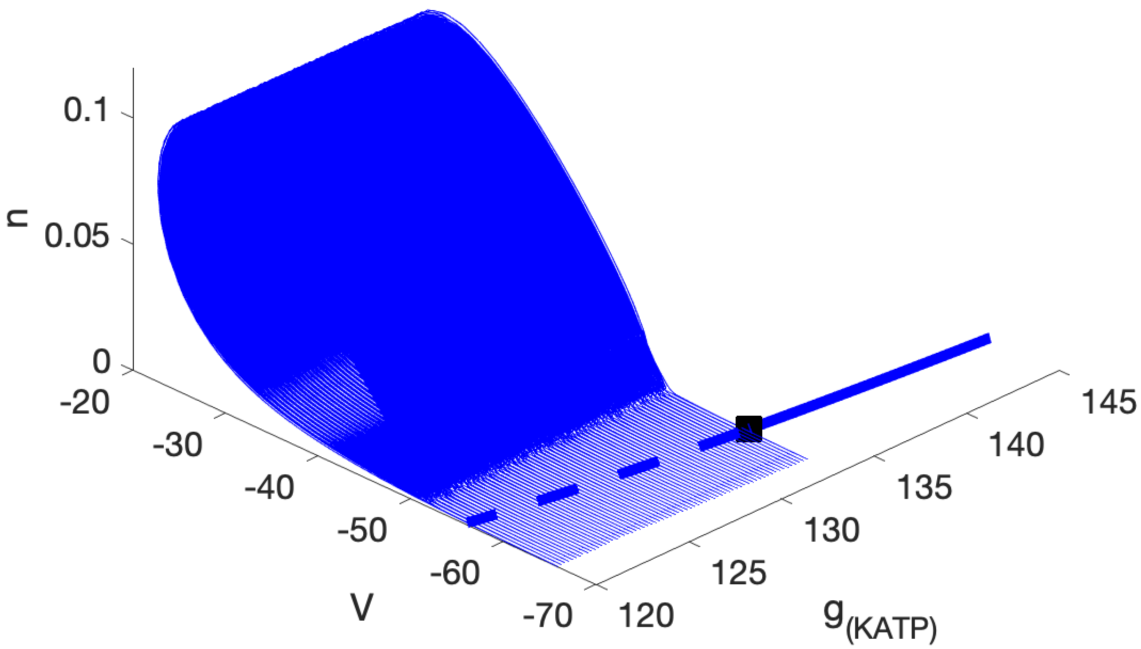

S3 Fig: A bifurcation diagram for the single cell model with the sub-critical Hopf bifurcation (black square) at $g_{K(ATP)} = 133$, which leads to the oscillatory behavior of an active cell after a limit point of cycles. The bistable range is so narrow that it is not shown here. Thick solid blue curve is the stable equilibrium. Thick dashed blue curve is the unstable equilibrium. Thin blue curves are the stable oscillations.

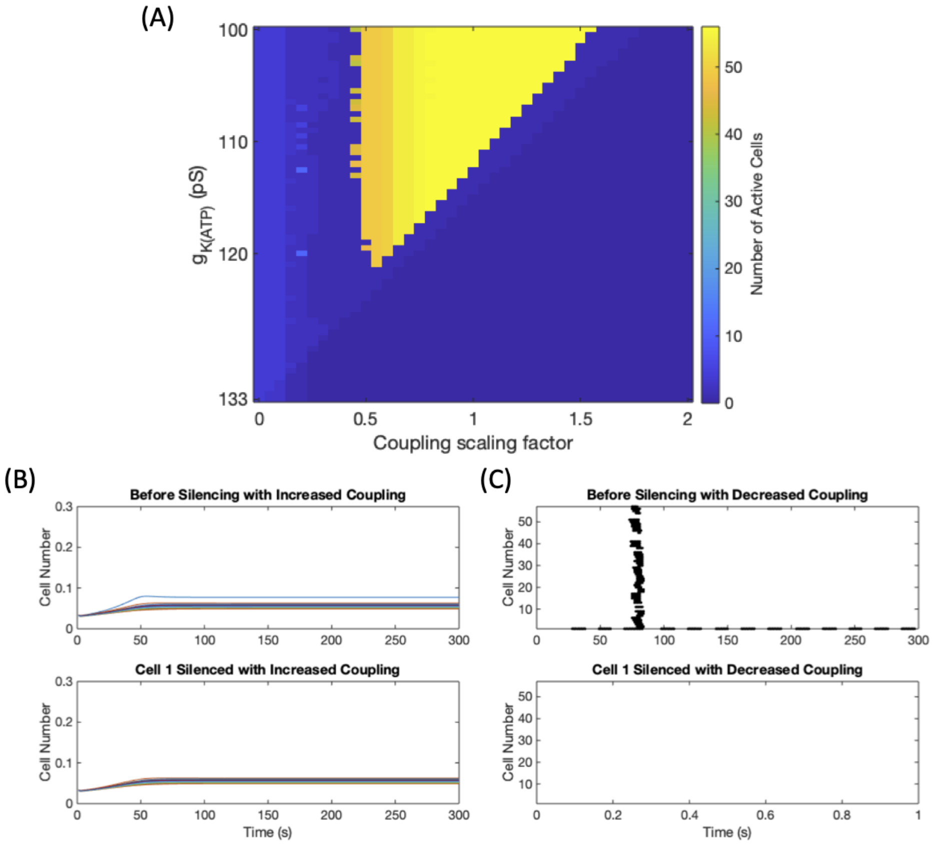

S4 Fig: (A) A heat map summarizing the variants of the predetermined "hub" cell studies.The $g_(K(ATP)$ of the predetermined "hub" cell was varied from 100 to 133 pS while the gap junctional coupling matrix was multiplied by a scaling factor between 0 and 2. The result is either all silent cells (dark blue), only the predetermined "hub" cell or the predetermined "hub" cell and its nearest neighbors active (lighter blue), or almost all cells active (yellow). (B) Calcium traces before (top) and while silencing (bottom) the predetermined "hub" cell for the islet with $g_{K(ATP)}$ of the predetermined "hub" cell set to 100 pS and the coupling matrix scaled by a factor of 2. (C) The raster plots for before (top) and while silencing (bottom) the predetermined "hub" cell for the islet with $g_{K(ATP)}$ of the predetermined "hub" cell set to 100 pS and the coupling matrix scaled by a factor of 0.5.

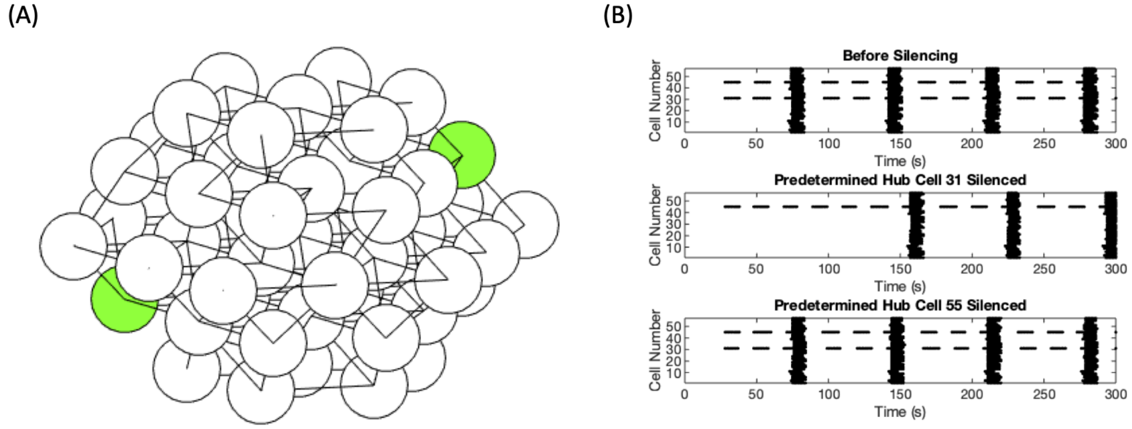

S5 Fig: An islet with two predetermined "hub" cells on opposing sides of the islet. (A) Islet schematic with the predetermined hub cells in green and the gap junction connections depicted with black lines. (B) The raster plots for (top) before silencing, (middle) silencing of one predetermined "hub" cell, and (bottom) silencing of the other predetermined hub cell.

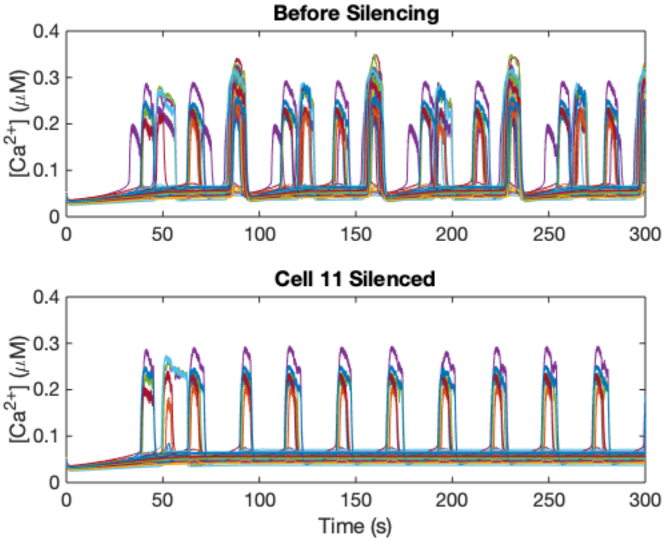

S6 Fig: The calcium traces before (top) and while silencing (bottom) corresponding to Fig 6(C), where this islet had the parameter distributions $g_{K(ATP)} \sim N(145, 14.5)$ pS and $g_c \sim N(5, 2.5)$ pS and cell 11 was a switch cell.

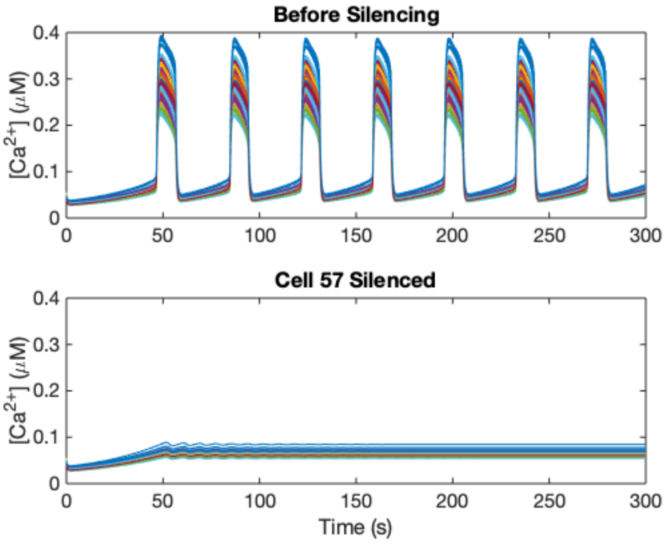

S7 Fig: The calcium traces before (top) and while silencing (bottom) corresponding to Fig 7(C), where this islet had the parameter distributions $g_{K(ATP)} \sim N(135, 13.5)$ pS and $g_c \sim N(200, 100)$ pS and cell 57 was a switch cell.

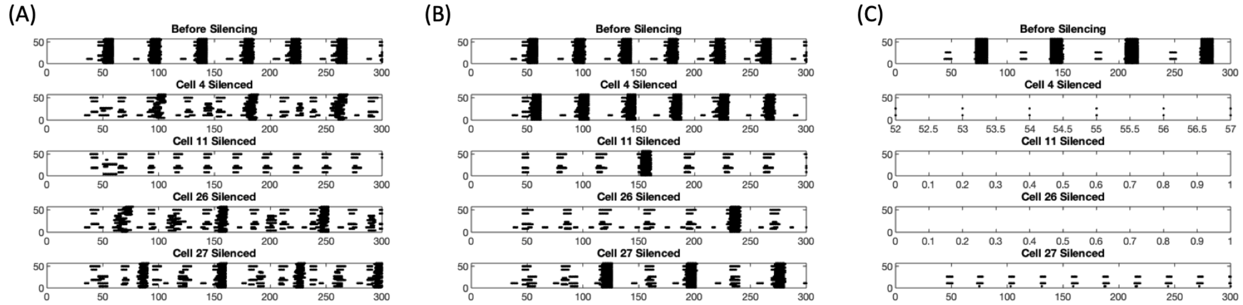

S8 Fig: The impact of increasing gap junctional conductance on the number of switch cells. (A) The raster plots for the parameter set in Fig 6. (B) The raster plots for the same cellular parameters, but with the gap junctional conductance multiplied by a factor of 2. (C) The raster plots for the same cellular parameters, but with the gap junctional conductance multiplied by a factor of 5.

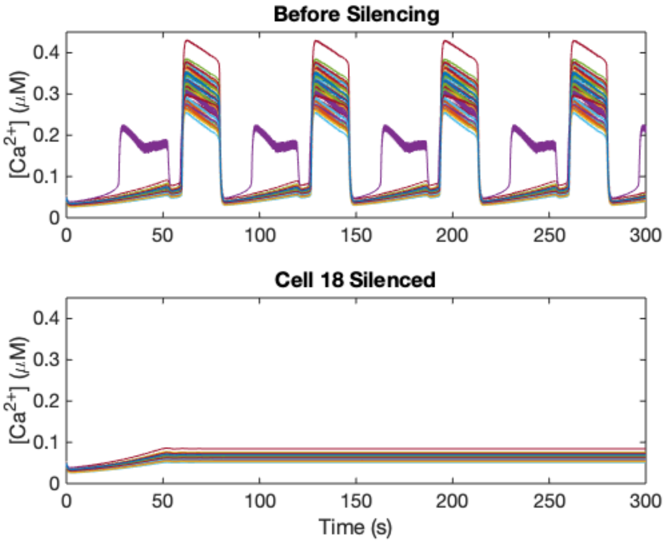

S9 Fig: The calcium traces before (top) and while silencing (bottom) corresponding to Fig 8(F), where the islet had spatially distributed $g_{K(ATP)}$ and g_{Ca} and cell 18 was a switch cell.

Silencing cells is not the same as removing them

Cell silencing has been used experimentally to temporarily remove cells from an islet to measure the impact of that cell on the network [1] rather than cell ablation that completely removes a cell from the network [2]. Through computational methods, a cell can be easily removed and added back into a network. By killing all of a cell's gap junction connections, it could no longer affect its neighboring cells and was effectively removed from the network. Thus, we briefly studied the differences between the two techniques computationally. In many cases, isolating switch cells found through silencing also led to a loss of activity in the islet. However, there were some cases in which isolating switch cells would have little impact on the islet bursting. Examples for low coupling ($g_c \sim N(5, 2.5)$) and high coupling ($g_c \sim N(200, 100)$) are shown in S10(A) and S10(B) Fig, respectively. As the coupling was increased from 10 pS to 200 pS with $g_{K(ATP)} \sim N(135, 13.5)$ pS, the percentage of parameter sets with silencing switch cells increased faster than the percentage of parameter sets with isolating switch cells. These results can be seen in S10(C) Fig. These differences can be accounted for by the hyperpolarizing current silencing the rest of the islet through coupling. As such, the stronger coupling leads to a larger gap between silencing and isolating.

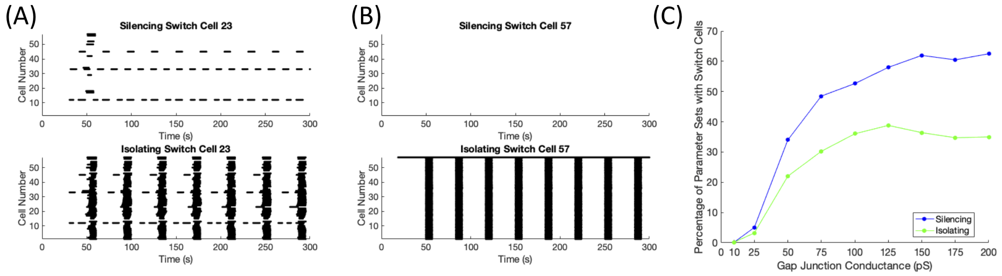

S10 Fig: **Silencing Versus Isolating.** (A) A parameter set with $g_c \sim N(5, 2.5)$ and $g_{K(ATP)} \sim N(145, 14.5)$ with the switch cell silenced (top panel) and the switch cell isolated (botton panel). (B) A parameter set with $g_c \sim N(200, 100)$ and $g_{K(ATP)} \sim N(135, 13.5)$ with the switch cell silenced (top panel) and the switch cell isolated (botton panel). (C) The percentage of parameter sets with silencing (blue) and isolating (green) switch cells out of the number of active parameter sets as the gap junction was increased with $g_{K(ATP)} \sim N(135, 13.5)$ pS.

References

[1] Natalie R Johnston, Ryan K Mitchell, Elizabeth Haythorne, Maria Paiva Pessoa, Francesca Semplici, Jorge Ferrer, Lorenzo Piemonti, Piero Marchetti, Marco Bugliani, Domenico Bosco, et al. Beta cell hubs dictate pancreatic islet responses to glucose. *Cell Metabolism*, 24(3):389–401, 2016. PMID: 27452146.

[2] Victoria Salem, Luis Delgadillo Silva, Kinga Suba, Eleni Georgiadou, S Neda Mousavy Gharavy, Nadeem Akhtar, Aldara Martin-Alonso, David CA Gaboriau, Stephen M Rothery, Theodoros Stylianides, et al. Leader β-cells coordinate ca^{2+} dynamics across pancreatic islets in vivo. *Nature Metabolism*, 1(6):615, 2019.
